# Does Smartphone Use Affect a Subsequent Swimming Training Session? Preliminary Results in Amateur Triathletes

**DOI:** 10.3390/s23135837

**Published:** 2023-06-23

**Authors:** Claudio Quagliarotti, Vittorio Coloretti, Emanuele Dello Stritto, Sarah Cuccurullo, Jessica Acalai, Romuald Lepers, Silvia Fantozzi, Matteo Cortesi, Maria Francesca Piacentini

**Affiliations:** 1 Department of Movement, Human and Health Sciences, University of Rome ‘Foro Italico’, 00135 Rome, Italy; claudruns@hotmail.it (C.Q.); e.dellostritto@studenti.uniroma4.it (E.D.S.);; 2 Department for Life Quality Studies, University of Bologna, 40126 Bologna, Italy; vittorio.coloretti2@unibo.it (V.C.); m.cortesi@unibo.it (M.C.); 3 INSERM UMR 1093-CAPS, UFR des Sciences du Sport, Université de Bourgogne, F-21000 Dijon, France; romuald.lepers@u-bourgogne.fr; 4 Department of Electrical, Electronic and Information Engineering, University of Bologna, 40136 Bologna, Italy; silvia.fantozzi@unibo.it

**Keywords:** mental fatigue, swimming kinematics, fatigability, motor coordination, warm-up

## Abstract

To date, the literature has failed to individuate a clear motivation for the performance decrement after a mental fatigue-inducing task. This study aimed to evaluate biomechanical and perceptual variables during a swimming training session in different mental fatigue states. Seven amateur triathletes watched a documentary, utilized a smartphone, or performed an AX-CPT for 45 min randomly on three different days. After, they performed a 15-min warm-up followed by 6 × 200 m at constant pre-set speed plus one 200 m at maximal effort. The mental fatigue status was assessed by the visual analog scale (VAS) and short-Stroop task results before, post-mental task, and post-swimming session. The biomechanical and motor coordination variables during swimming were assessed using five IMU sensors and video analysis. The heart rate and rate of perceived exertion were monitored during the task. No differences in biomechanical and perceptual variables were found between and within conditions. Higher mental fatigue was found only in the AX-CPT condition at post task by VAS. In this preliminary study, no changes in swimming biomechanics were highlighted by mental fatigue, but the warm-up performed may have counteracted its negative effects. Further studies are recommended.

## 1. Introduction

Mental fatigue is a psychobiological state that arises during prolonged, demanding cognitive activity and results in an acute feeling of tiredness and/or decreased cognitive ability [1,2,3]. The exact mechanisms generated by mental fatigue remain unknown to date [1]. In general, a decrease in general arousal and alertness [4,5,6,7], with a decrease in modulation in the sensory process [6,8], cognitive control [9,10], and attention [11,12], have all been recognized as primary factors affecting subsequent performance. The increase in adenosine concentration in the prefrontal cortex suggests a change in executive function and behaviors, while the higher activity found in the insula and cingulate cortex could affect the translation of afferent signals in sensation, modifying, for example, the rate of perceived exertion (RPE) [13,14]. Physical performance has been shown to be negatively impacted by prior mental fatigue [1,3,13]. An increased risk of error, decreased emotion regulation, and a reduction of sensorimotor function—and thus the ability to initiate and stop movement, monitor and change behavior, and plan subsequent moves—have been reported [1,13]. 

Although mental fatigue has shown no effect on oxygen consumption, ventilation, blood lactate, heart rate, stroke volume, or arterial pressure [1,15], a decrease in endurance performance has been well established [1,3,13,16]. The negative impact on endurance performance was explained by the higher RPE reported throughout the effort or by a possible change in the pacing strategy adopted. However, contradicting results on this issue have been reported [15,17] and no differences in the pacing strategy adopted were found [17,18,19]. In particular, after mental fatigue induction, a decrease in swimming performance has been shown at distances equal to or longer than 100 m, both in the acute (just one training session) [17] and in the long term (periodic use before each training session) [20]. Moreover, the analysis of the pacing adopted by the swimmers during the bout revealed no differences in time during the first 50 m of a 100 or 200 m, but a negative impact on time was highlighted in the following laps [17]. However, to the best of our knowledge, no study to date evaluated the effects of a mental fatigue status on biomechanics variables in a cyclic activity such as swimming. Changes in biomechanical variables, due to the sensorimotor function reduction [1,13], could explain the performance impairment in an endurance task. Indeed, the synergic action of the body segments during swimming is critical to maximizing propelling proficiency and minimizing drag, leading to a more economical energy cost of swimming over a given distance [21]. Recently, an integrated analysis of stroking, breathing, and kicking in front crawl swimming was validated utilizing five wearable inertial sensors [22]. This procedure would permit the provision of information about the changes in swimming motor coordination due to specific conditions that athletes may experience during training, as recently shown in a study with a similar protocol [23].

One of the most commonly utilized cognitive tasks to induce mental fatigue is the AX-Continuous Performance Test (AX-CPT) [1,2,15,24]. The utilization of an AX-CPT for 45 min has been shown to induce a mental fatigue status for at least the following 60 min [24]. The assessment of mental fatigue status can be conducted by different methods, divided into objective (i.e., physiological), subjective (i.e., perceptual), and task performance evaluations. However, due to the limitations of objective evaluation (such as electroencephalography), subjective methods such as the utilization of a visual analog scale (VAS) have been suggested as the most practical [24]. However, differences in persistence and characterization of mental fatigue have been shown [25,26]. In addition to the direct assessment, the assessment of mood changes seems important to confirm that the alteration found would be directly attributed to the high cognitive load and not to the monotony of the task [2,13]. Recently, it has been shown that mental fatigue, as assessed by the results of a short-Stroop task, could also be induced by the utilization of a smartphone, which has a negative effect on physical performance [17,20,27]. These findings are very intriguing from a practical point of view due to the massive utilization of smartphones in our daily lives.

The aim of this study was to evaluate the possible effects of mental fatigue status on biomechanical and perceptual variables during a swimming training session. We hypothesized that a change in swimming motor coordination after a mentally fatiguing task would negatively impact swimming performance. 

## 2. Materials and Methods

### 2.1. Participants

Seven male amateur (Tier 2 [28]) triathletes were recruited for the study. All triathletes were familiarized with the rate of perceived exertion scale (CR-10 modified, Italian version [29]), commonly utilized to monitor training load and widely promoted by the Federation during training courses for coaches. Detailed information about triathletes is provided in Table 1. Information regarding procedures was provided to each participant; written informed consent and personal information treatment were obtained. The study was approved by the Institutional Review Board (CAR 123/2022) and in accordance with the principles of the Declaration of Helsinki.

### 2.2. Design

**Figure 1:**
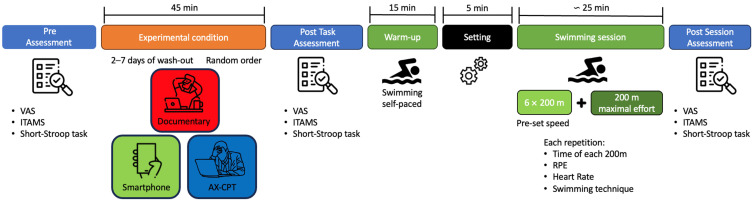
Study design.

Each triathlete participated in three test sessions performed on three separate days that differed only in the stimulation provided to induce mental fatigue. The design is summarized in Figure 1. The conditions of each session (i.e., documentary, smartphone, and AX-CPT) were performed in a random and counterbalanced order using a computer-generated randomization order. The sessions were performed just before the competitive season, at the same time of day, separated by at least 48 h and no more than 7 days apart. The participants were instructed to be well hydrated, to maintain similar eating, sleeping, and training habits, and to avoid intensive exercise (for 48 h), alcohol (24 h), caffeine (4 h), nicotine (4 h), and continuous use of their smartphone (3 h) before the tests. 

### 2.3. Methodology

1. *General and environmental information*

Before the first test session, triathletes filled out an online survey (Google Form, Google, California, Mountain View, USA) to collect individual information such as age, height, and training data (see Table 1). The tests were performed in an indoor pool (length: 33.33 m, water temperature = 27.5 ± 0.2 °C, air temperature = 30.4 ± 3.9 °C) traditionally used by the triathletes. Before each test session, body mass and fat were estimated by an impedance balance (Mi Body Composition Scale 2, Xiaomi, Beijing, Haidian District, China).

2. *Conditions—Mental fatigue induction*

The manipulation consisted of a continuous performance test of 45 min where triathletes sat in a chair in front of a table, wearing a headset, in a separate quiet location near the swimming pool (~40 m). An operator was always present near the triathlete to assess the correct manipulation procedure. The task was different for each test session, randomly selected between AX-CPT, smartphone use, and documentary watching. 

The AX-CPT consists of sequences of letters visually presented (one at a time in continuous) on a 13-inch laptop screen [15,24]. Each sequence was composed of four letters, presented once at a time, the first and last as cues colored in red, and two distractor letters in the middle colored in white. Each letter was presented centrally on a black background for a duration of 300 ms in 24-point uppercase Helvetica font and was followed by a 1200-ms interval. The cue letters could be any letter except for K and Y, while the distractor letters could be any letter except for A, K, X, and Y. Triathletes were instructed to press the right button (letter “I” in the keyboard QWERTY-type) after a sequence containing A as the first letter and X as the second letter as a cue, with any distractor letters between. Otherwise, they were instructed to press the left button (letter “E”). Letter sequences were presented in pseudorandom order, for a total of 450 sequences, such that target (AX) trials occurred with 70% frequency and nontarget trials occurred with 30% frequency. Nontargets were divided evenly (10% each) among the following trial types: BX trials, in which an invalid cue (i.e., non-A) preceded the target; AY trials, in which a valid cue was followed by a nontarget probe (i.e., non-X); and BY trials, in which an invalid cue was followed by a nontarget probe. Any missed or incorrect response elicited a beep sound from the headset as a prompt to increase speed and accuracy. Performance was scored automatically by the computer based on correct responses and response time. The proportion of correct responses to the AX trials and reaction time of the AX-CPT were compared in each 15-min period as a manipulation check [15]. The AX-CPT was created by free online software (PsyToolkit V.3.4.0) [30,31]. The triathlete was instructed by an oral description of the task procedure and a short instruction guide presented on the computer screen. Moreover, just before the start of the manipulation, a short version of AX-CPT containing five pseudorandom sequences was performed. The test started only when the triathlete was confident with the test procedure.

In the smartphone condition, each triathlete was instructed to utilize his own smartphone, watching the screen for the entire duration of the manipulation [17]. The triathlete was allowed to utilize any application installed with the possibility to switch it at her preference. The only request was not to watch any long videos (i.e., >2–3 min) or listen to music. 

The documentary condition consisted of watching an entire documentary video. The triathlete was allowed to select the video for inclusion in two different documentary series (*Predators Bloodlines*, National Geographic UK, 2020; *Japan—Between Earth and Sky*, National Geographic UK, 2018; both available on Disney+). The videos were chosen based on their content being engaging and capable of maintaining a neutral mood, as identified during pilot testing performed by at least two individual operators [24].

3. *Mental fatigue assessment*

The assessment of the mental fatigue status was provided (in the following order) by the utilization of the VAS for mental fatigue and motivation, a short-Stroop task, and the Brunel mood scale. The assessment measures were performed at the beginning of the session (pre), immediately after the conditioning task (post-task), and as soon as possible after the swimming session (post-session).

The VAS consisted of a line (20 cm) presented at the center of a white sheet of paper. At the extremities of the line, there was a little vertical line (one for each extremity), with a label just beneath: “No mental fatigue” or “No motivation” on the left side and “Maximal mental fatigue” or “Maximal motivation” on the right. The triathlete was instructed to sign his momentary perceived mental fatigue or motivation with a vertical line with a pen [17,20,24,25,26,32].

The short-Stroop task consisted of a short version of the Stroop task (30 words) in the Italian version, performed on a 13-inch laptop screen [17,20,27]. The Stroop task consists of a sequence of words indicating a color (i.e., red, green, blue, and yellow) colored with a color corresponding at once to the possible color indicated. All words were presented centrally, on a black background, for a duration of 500 ms in 24-point uppercase Helvetica font, followed by a 2000-ms interval. The triathlete was instructed to answer as quickly and accurately as possible by pressing the colored button corresponding to the color of the word presented. The fingers of the left hand were positioned on the A (equal to the color red) and D (green) buttons, and the fingers of the right hand on the J (blue) and L (yellow) buttons, on a QWERTY keyboard. After an answer was given, or after 2000 ms without an answer, a white text reporting “correct answer” or “incorrect answer” was presented at the center of the screen. The stimuli were presented in random order for a total of 30 words. Performance was scored automatically by the computer based on correct responses and response time. The short-Stroop test was created by free online software (PsyToolkit V.3.4.0) [30,31]. The triathlete was instructed by an oral description of the task procedure and a short instruction guide presented on the computer screen. Moreover, just before the start of the manipulation, a trial of the short-Stroop test was performed (~5 words). The test was started only when the triathlete was confident with the test procedure.

The Italian version of the Brunel Mood Scale (Italian Mood Scale, ITAMS [33]) was utilized to evaluate the momentary mood perceived by the triathletes. The triathlete was invited to fill out an online ITAMS (Google Form, Google, California, Mountain View, USA) utilizing a 13-inch laptop. The ITAMS consists of 24 items, including 6 subscales corresponding to the underlying factors of anger, confusion, depression, fatigue, tension, and vigor. For each item, triathletes select a verbal answer corresponding to a score from 0 to 5. The mood items for each subscale are summed. To account for the ephemeral nature of the mood construct, the ITAMS asks triathletes to rate how they feel right now.

4. *Swimming session*

A standard warm-up, consisting of self-paced swimming for up to 15 min [34,35], was performed before each swimming test. The swimming test protocol for a 7 × 200 m front crawl consisted of 6 repetitions at a constant speed and the last one at maximal effort, with 30 s rest between repetitions. The constant speed was selected based on the individual average race speed of the Olympic distance triathlon (1500 m) and/or corresponding swimming intensity training at threshold [36] (see Table 1). A sound pacer (Tempo Trainer, Finis, Massa, Italy) was placed inside the swimming cap, and the swimmer followed the audio signal to synchronize with his pre-set speed. The time performance of each length was recorded by at least one expert investigator (FINIS 3X-300M, FINIS, Inc., Livermore, CA, USA) and by video recordings. The subjects with a time difference in at least one repetition >3% were discarded and not analyzed because of possible differences in biomechanical variables, as previously reported [37]. The pacing adopted during the last 200 m at maximal effort was estimated from the time performance of each length (6 × 33.33 m) expressed as a percentage of the total average speed. 

Five wearable inertial unit sensors (WaveTrack Inertial System waterproof, Cometa, Milan, Italy, 128 Hz, accelerometer full scale: 16 g, gyroscope full scale: ±2000°/s) were placed on the occipital bone, on the wrists, and 1 cm above the lateral malleolus. The sensors were fixed with two swimming caps on the head and with biadhesive/co-band tape on the limbs. The average value of the following biomechanical variables was calculated for each 200-m repetition by the gyroscope signal output of each sensor [22]: breathing count (total, right and left side); timing of breathing (left and right) with respect to stroke cycle duration, starting with the hand entry; time of breathing action; strokes count/lap (right and left side); stroke length (SL) per lap (right and left side); time of stroke action (right and left side); kicks count/lap (right and left side); timing of kicks (first, second, and third, when conducted) with respect to stroke cycle duration, starting with the hand entry; time of kick action (right and left side); stroke index (SI) (right and left side); and Index of Synchronization (IdS). For more detailed information about the set-up and analysis of sensor data, we recommend referring to the article published by Fantozzi et al., 2022 [22].

The test was recorded by an underwater camera (Hero4 Black, 120 Hz, GoPro, California, San Mateo, USA, ) placed in the sagittal plane of the swimmer. At least one to three complete stroke cycles were recorded each time the participant passed in front of the camera. Kinovea software version 0.8.15 (Joan Charmant & Contrib.) was used to manually analyze frame-by-frame the video sequences. The arm stroke phase events (entry, pull, push, and recovery) were identified using video analysis to estimate the stroke phase percentages, the stroke rate, and the index of coordination (IdC) [38]. These variables were presented as the mean value of the first and seventh repetitions of each test.

HR was continuously recorded (HRM-Tri and Forerunner 935, Garmin, Kansas, Olathe, USA) during the test. The mean value was estimated for each repetition and expressed as the percentage of the maximal heart rate (%HR_max_). After each 200 m, the rate of perceived exertion (RPE) was collected (CR-10 modified scale, Italian version [29]).

### 2.4. Statistical Analysis

The statistical package SPSS version 25.0 (IBM, Chicago, IL, USA) for Windows OS was used for statistical analysis. To evaluate differences between conditions, the non-parametric Kruskal–Wallis H test was performed for all variables. A Wilcoxon test was performed post hoc, both with biserial correlation (r) as effect size, to assess pairwise differences between the conditions (documentary, AX-CPT, and Smartphone).

To evaluate differences within the condition, the non-parametric Friedman test, both with Kendall W as effect size, was performed for all variables. A Mann–Whitney U test was performed post hoc, both with biserial correlation (r) as effect size, to assess pairwise differences within the condition between each repetition with the first one (five times) or each time of metal fatigue status assessment (three times per VAS, ITAMS, and Stroop task). The value of r was considered small (0.100–0.299), moderate (0.300–0.499), large (0.500–0.699), very large (0.700–0.899), and extremely large (≥0.900) [39]. The significance level was set at *p* ≤ 0.05. Data are presented as median ± interquartile range.

## 3. Results

1. *Swimming session*

**Figure 2:**
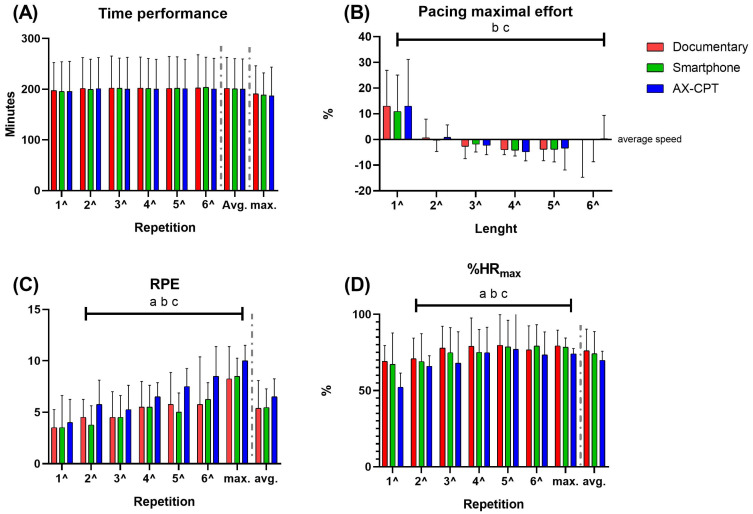
The performance time (**A**), rate of perceived exertion (**C**), and percentage of maximal heart rate (**D**) during each 200 m and the average of all 7, and the pacing adopted during the maximal repetition (**B**) in each condition. a, b, and c are significantly different within the documentary, smartphone, and AX-CPT conditions, respectively.

Three of the recruited triathletes achieved a time difference of >3% in at least one of the pre-speed set repetitions (the first three subjects in Table 1). These three subjects were discarded and not analyzed, as previously stated. The analyzed triathletes completed the first six repetitions at a pre-set speed with a mean time difference of 0.00 ± 1.17% and performed the maximal repetition significantly faster than the previous repetitions. No time performance difference was found between or within conditions in the first six 200 m. Regarding the last 200 m (maximal), no difference between conditions was found (Figure 2A). The pacing adopted during the maximal repetition was significantly different within smartphone (*p* = 0.018) and AX-CPT (*p* = 0.005) conditions, but the post hoc analysis did not confirm these differences (all *p* > 0.050). Meanwhile, no other differences were found within the documentary condition or between conditions (Figure 2B).

**Figure 3:**
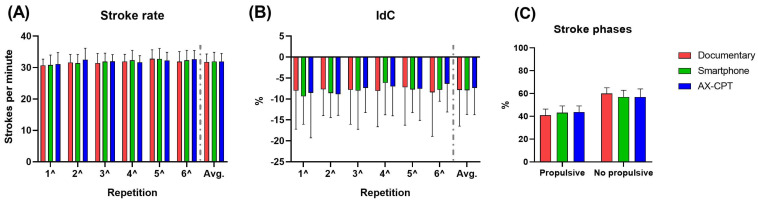
Stroke rate (**A**), Index of Coordination (**B**) during each 200 m and average of the first 6 repetitions, and the mean percentage of propulsive and non-propulsive stroke phases (**C**) in each condition. Data from video analysis.

**Figure 4:**
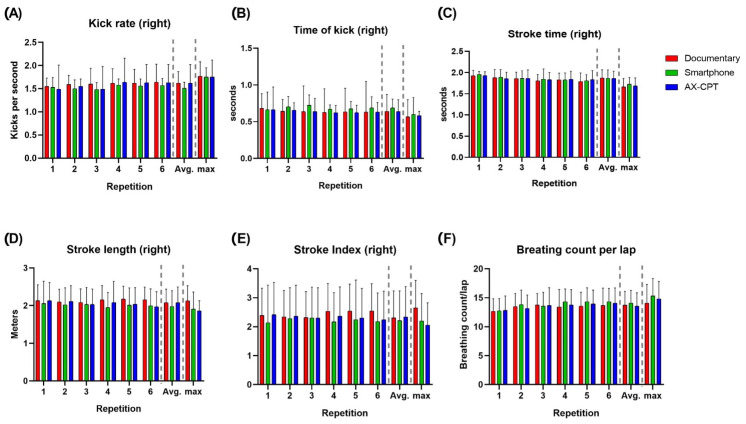
Kick rate (**A**), kick time (**B**), stroke time (**C**), stroke length (**D**), stroke index (**E**) of the right side, and breathing count per lap (**F**) during each 200 m, average of the first 6 repetitions and the last maximal repetition in each condition. Data from wearable sensors.

A difference was found within each condition in both RPE and %HR_max_, but none was confirmed by post hoc analysis. Moreover, no difference between conditions was found for RPE and %HR_max_ (Figure 2C,D). No difference was found between or within conditions in biomechanical parameters (in Figure 3, variables from video analysis and in Figure 4, some variables from inertial sensors as examples).

The detailed data and statistical analysis results are provided as Supplementary Files.

2. *Mental fatigue*

**Figure 5:**
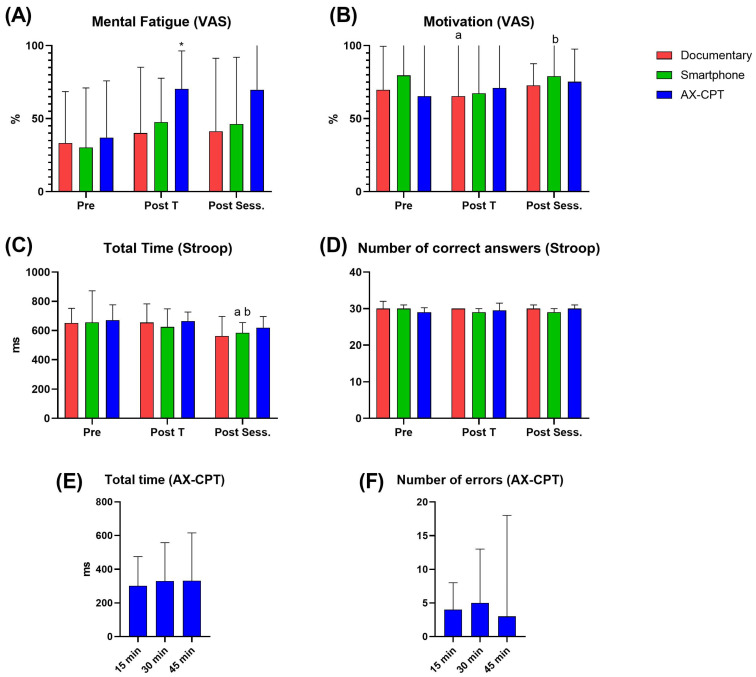
Mental fatigue and motivation assessment during pre-, post-task (Post T), and post-session (Post Sess.) by Visual Analogue Scale (VAS) (**A**,**B**), short-Stroop task results (**C**,**D**), and during the AX-CPT (**E**,**F**). * Significantly different from the Documentary condition, a—Significantly different from pre, b—Significantly different from post task.

The analysis of mental fatigue assessment utilizing VAS showed significantly higher perceived fatigue during the post-task evaluation in the AX-CTP condition compared only to the documentary (*p* = 0.002) (Figure 5A). Mental fatigue assessment utilizing the results of the short-Stroop task showed a shorter reaction time within the smartphone condition in the post-session compared to both pre- and post-task (*p* < 0.005) (Figure 5C,D). Motivation, assessed with the VAS, showed a significant difference within the documentary condition between pre- and post-task (*p* = 0.028) and within the smartphone condition between the post-task and post-session (*p* = 0.028) (Figure 5B). The results of performance during the AX-CPT did not show a difference in the reaction time, the number of correct answers, or the reaction time of correct answers. In the mood assessment, no differences were highlighted between pre-, post-task, and post-session within each condition per subscale, except in the documentary condition between post-task and post-session in the vigor subscale (*p* = 0.017).

The detailed data (S1) and statistical analysis (S2) results are provided as Supplementary Files.

## 4. Discussion

The aim of this study was to evaluate the possible effects of mental fatigue on biomechanical and perceptual variables during a swimming training session. Contrary to our expectations, no differences were found between conditions for all analyzed variables (Figure 2, Figure 3 and Figure 4). Indeed, previous studies showed that mental fatigue negatively affected a subsequent cyclic endurance task [1,3,13,16], but the motivations for this decline are not justified by both physiological and perceptual variables. We hypothesized that a reduction in sensorimotor function [1,13] could affect swimming biomechanics and thus negatively affect performance. The adoption of a pre-set speed permits us to directly evaluate the changes in physiological, perceptual, and biomechanical variables between the different conditions, while the last maximal repetition allows us to evaluate changes in pacing strategy and performance. These results could be explained by multiple factors. It has been hypothesized that mental fatigue could affect more an isolation task (i.e., hand grip) requiring a high proportion of direct corticospinal projection compared to a whole body task (i.e., cycling) primarily controlled by automatic motor processes through central pattern generators [40]. However, we hypothesized that a minimal change in swimming kinematics induced by mental fatigue could, at least partially, explain the decrease in performance experienced by athletes.

Another factor that has to be taken into account is the possible effect of the warm-up protocol performed by our triathletes, which could have decreased the effects of mental fatigue. In the present study, triathletes performed a warm-up of 15 min before the 7 × 200 [41], just between the mental fatiguing task and the training session. Similarly, a decrease in mental fatigue status was reported following 15 min of cycling at moderate intensity [25]. The authors hypothesized that it was probably due to the increased dopamine secretion (and arousal) during exercise that can counteract the effects of mental fatigue [25]. We can hypothesize that the warm-up decreased the magnitude of the negative effects of mental fatigue that would have normally been evident without the warm-up. Previous studies that evaluated the effects of mental fatigue on swimming performance did not provide information on the warm-up protocol [18,20] or performed a short warm-up of five minutes, including a 5 × 10 m sprint followed by five minutes of rest [17]. Thus, it could be hypothesized that a 15-minute warm-up could minimize the effects of mental fatigue on performance, but further studies on the topic are needed.

We used two different methods to induce mental fatigue: the classical AX-CPT and the more ecological smartphone use. Mental fatigue was then assessed by the VAS and by a short Stroop task. It has been previously suggested that using a smartphone could induce a mental fatigue status and that mental fatigue could be assessed by the results of short tasks, such as the Stroop [17,20,27]. To the best of our knowledge, no direct comparisons of different methods to induce mental fatigue, including smartphone use, have been reported. Nevertheless, our results failed to confirm these findings. We found a higher perceived mental fatigue only after the execution of the “classic” AX-CPT, compared to the control condition (documentary) (Figure 5). It could be reasonable to affirm that intense smartphone use would induce a “lighter” perceived mental fatigue status because of the lack of difference between this condition and the two others (both *p* = 0.128). The subjects were instructed to normally utilize their smartphones for 45 min, watching the screen continuously and avoiding videos. Thus, compared with a “classic” mental fatigue-inducing task, such as the AX-CPT utilized in this study, the subjects were allowed to switch between different applications, and thus cognitive tasks, at their preference. Moreover, the possibility to switch the cognitive task according to preference, modifying both the duration and type of stimulus according to the momentary individual attitude, could, on the one hand, elevate the engagement on the task but also decrease the “cognitive load” enforced. In the assessment of mental fatigue status, several investigations utilized the responses during a cognitive task, such as the increase in reaction time and the number of errors, as indicators of an induced mental fatigue status [15,24]. However, our results showed a discrepancy between VAS, indicating higher perceived mental fatigue in the AX-CPT condition compared to the control, and the results of the short-Stroop task, highlighting no differences (Figure 5). It would be reasonable to affirm that performing an AX-CPT for 45 min would induce mental fatigue, as already documented in the literature [1,15,24]. Although mental fatigue assessment by subjective measures like VAS is questionable compared to objective ones [26], the lack of changes in the results of the short-Stroop task was an unexpected result and made us inquire about the sensitivity of such methods to detect mental fatigue.

## 5. Practical Applications, Limitations, and Future Perspectives

No differences in perceptive, physiological, and biomechanical variables were found during a 7 × 200 m swimming training session after a prolonged cognitive task. It could be hypothesized that the warm-up performed immediately after the mentally fatiguing task could counteract and minimize the negative effects of a mental fatigue status on endurance performance. Moreover, the results of the present study did not confirm the use of a smartphone as a mental fatigue-inducing task when compared to a classic mental fatigue status-inducing task (AX-CPT) or the use of the short-Stroop task to assess mental fatigue compared to a subjective measure (VAS). However, further studies are needed to confirm these statements.

This study has some limitations. The main limitation is the low number of triathletes recruited and the fact that they were all amateurs. Moreover, three subjects were also discarded for the swimming performance analysis because they did not perform the pre-set speed repetitions correctly. Despite this, it is important to note that the data results showed very strong agreement in each variable analyzed, both between and within conditions. Further studies should consider expanding the sample size to include other athletes, such as pool swimmers or elite triathletes.

From a practical point of view, using a smartphone for 45 min consecutively, without distraction, does not correspond to what is normally carried out by athletes before starting a training session. This timing was selected following the results presented in the literature indicating a time of 45 min of AX-CPT to induce a persistence of mental fatigue status for more than 1 h [24]. Moreover, the mental fatigue status was assessed by a subjective measure, while a better characterization would be obtained by objective measures [26]. Other studies evaluating methods to induce and assess a mental fatigue status and its components, such as timing, persistence, type, and sequence of tasks, are necessary for a proper induction, on the one hand, and for the evaluation of possible risk behaviors that could be adopted by athletes before their training sessions, on the other.

Finally, a sound pacer was provided to the triathletes to help them correctly perform the pre-set swimming speed and thus evaluate the effects of each condition on the performance variables. However, during a real training session, the athletes do not utilize a sound pacer.

## Figures and Tables

**Table 1:** Subjects’ characteristics, training information, and weekly screen time.

ID	Sex	Age (Yrs)	Height (cm)	Body Mass (Kg)	Body Fat (%)	Triathlon Distance Performed	Mean Speed at Threshold (m/s)	Years of Triathlon Practice	Triathlon Training Volume (h/wk)	Swimming Practice (Yrs)	Swimming Training Volume (h/wk)	Swimming Training Volume (Km/wk)	Swimming Training Volume (sessions/wk)	Weekly Screen Time
1	M	34	187	82.2	20.8	HF, F	0.93	6	10	6	3	8	3	15 h00
2	M	40	174	79.2	19.3	S, O	1.15	3	8	>10	5	11	4	24 h43
3	M	28	177	68.1	11.7	S	0.83	2	10	2	1	2	1	40 h23
4	M	26	178	73.9	15.2	S, O, HF	0.87	2	10	2	3	4	3	42 h38
5	M	21	178	74.7	16.6	SS, S, O	1.15	8	12	8	2	2	1	46 h11
6	M	25	176	69.9	13.2	O, HF, F	0.88	2	12	2	3	6	2	27 h07
7	M	21	176	62.6	6.1	S, O	1.25	2	25	>10	9	18	4	53 h24
**Tot**		27.9 ± 7.0	178 ± 4.2	72.9 ± 6.7	14.7 ± 5.0		1.01 ± 0.17	3.6 ± 2.4	12.4 ± 5.7	5.7 ± 3.7	3.7 ± 2.6	7.3 ± 5.7	2.6 ± 1.3	35 h38 ± 13 h38

M—Male; F—Female; SS—Super-Sprint; S—Sprint; O—Olympic; HF—Half-Full; F—Full; Screen Time—average weekly screen time on own smartphone; Yrs—Years; h/wk—hours per week; Km/wk—Kilometers per week.

## Data Availability

The data presented in this study are available upon request from the corresponding authors. The data are not publicly available due to privacy restrictions.

## Supplementary Materials

The following supporting information can be downloaded at: https://www.mdpi.com/article/10.3390/s23135837/s1, S1: detailed data; S2: statistical analysis.
