# Supplementary material for: Does Smartphone Use Affect a Subsequent Swimming Training Session? Preliminary Results in Amateur Triathletes
Source: Sensors (Basel). 2023 Jun 23;23(13):5837. doi: 10.3390/s23135837 (PMC10347285; doi:10.3390/s23135837)
Supplement: Supplementary file 1 [file sensors-23-05837-s001.zip › S1-Detailed data.pdf]

Detailed data

- **Mental Fatigue VAS**

	Pre	Post task	Post session
Documentary	33.3±35.2 %	40.1±45.1 %	41.4±50.0 %
Smartphone	30.2±40.8 %	47.5±30.2 %	46.3±45.7 %
AX-CPT	37.0±38.9 %	70.4±26.0 %	69.8±61.2 %

- **Motivation VAS**

	Pre	Post task	Post session
Documentary	69.8±29.7 %	65.4±35.8 %	72.8±14.8 %
Smartphone	79.6±45.1 %	67.3±53.7 %	79.0±32.7 %
AX-CPT	65.4±35.8 %	71.0±38.3 %	75.3±22.3 %

- **Short-Stroop task – Reaction Time**

	Pre	Post task	Post session
Documentary	651.47±100.87 ms	655.10±128.10 ms	562.87±134.47 ms
Smartphone	656.27±215.57 ms	624.37±124.70 ms	584.83±69.60 ms
AX-CPT	670.92±104.97 ms	665.82±61.36 ms	619.47±77.06 ms

- **Short-Stroop task – Number correct answers**

	Pre	Post task	Post session
Documentary	30±2	30±0	30±1
Smartphone	30±1	29±1	29±1
AX-CPT	29±1.3	29.5±2	30±1

- **Short-Stroop task – Reaction time of correct answers**

	Pre	Post task	Post session
Documentary	651.46±87.31 ms	655.10±128.10 ms	564.68±134.47 ms
Smartphone	657.48±176.03 ms	629.34±122.89 ms	581.24±68.24 ms
AX-CPT	671.40±90.15 ms	667.57±58.20 ms	623.81±77.06 ms

- **AX-CPT**

	15 min	30 min	45 min
Reaction Time	300.87±174.29 ms	329.21±228.33 ms	330.71±285.01 ms
Number of errors	4±4	5±8	3±15
Reaction time of correct answers	242.27±190.00 ms	323.25±219.30 ms	319.24±279.31 ms

- **ITAMS**

**Documentary**	Pre	Post task	Post session
Anger	0.00±2	0.00±2	0.00±2
Confusion	0.00±1	0.00±0	0.00±0
Depression	0.00±0	0.00±1	0.00±3
Fatigue	3.00±4	3.00±4	5.00±3
Tension	0.50±3	0.00±1	0.00±4
Vigor	9.50±6	8.00±7	11.00±2

**Smartphone**	Pre	Post task	Post session
Anger	0.00±2	0.00±0	0.00±2
Confusion	0.00±0	0.00±1	0.00±2
Depression	0.00±1	0.00±1	0.00±1
Fatigue	3.00±1	4.00±2	2.00±4
Tension	1.00±1	0.00±1	0.00±1
Vigor	9.00±5	8.00±4	8.00±3

**AX-CPT**	Pre	Post task	Post session
Anger	0.00±3	1.00±4	1.00±3
Confusion	0.50±4	3.00±3	1.00±3
Depression	1.00±4	0.00±1	0.00±1
Fatigue	4.00±5	6.00±6	6.00±3
Tension	2.00±6	2.00±2	0.00±4
Vigor	9.00±3	8.00±3	8.00±6

- **Time performance (s)**

	1st 200 m	2nd 200 m	3rd 200 m	4th 200m	5th 200m	6th 200m	Average of first 6 200m	7th 200m (maximal)
Documentary	197±55.35	201.72±60.54	202.04±63.31	202.01±61.46	201.56±62.84	202.35±65.64	201.65±61.05	190.76±55.29
Smartphone	195.56±58.44	199.56±59.60	201.81±59.61	201.48±59.07	201.96±61.91	203.58±59.51	200.84±59.50	188.36±43.80
AX-CPT	196.02±58.82	201.68±60.60	200.60±62.23	200.57±58.22	201.64±57.20	200.75±60.02	200.55±59.17	187.35±56.20

- **Pacing all-out (% of average speed)**

	1st lap	2nd lap	3rd lap	4th lap	5th lap	6th lap
Documentary	12.95±13.96	0.66±7.25	-2.72±4.74	-4.04±1.89	-3.82±4.46	-0.09±14.66
Smartphone	10.93±14.13	-0.31±4.38	-1.81±3.08	-4.16±2.29	-3.82±4.93	-0.08±8.60
AX-CPT	12.95±18.21	0.81±4.85	-2.31±3.63	-4.84±3.51	-3.45±8.43	0.31±9.07

- **RPE**

	1st 200 m	2nd 200 m	3rd 200 m	4th 200m	5th 200m	6th 200m	7th 200m (maximal)	Average of first 6 200m
Documentary	3.5±1.8	4.5±1.8	4.5±2.5	5.5±2.5	5.8±3.1	5.8±4.6	8.3±3.1	5.4±2.7
Smartphone	3.5±3.1	3.8±1.9	4.5±2.1	5.5±2.1	5.0±1.9	6.3±1.6	8.5±1.8	5.5±1.8
AX-CPT	4.0±2.3	5.8±2.4	5.3±2.4	6.5±1.4	7.5±1.8	8.5±2.9	10.0±1.5	6.5±1.8

- **Heart rate as %HRmax**

	1st 200 m	2nd 200 m	3rd 200 m	4th 200m	5th 200m	6th 200m	7th 200m (maximal)	Average of first 6 200m
Documentary	69.01±10.49	70.89±13.46	77.89±14.29	79.06±18.56	79.68±20.10	76.73±15.72	79.28±10.32	76.08±14.20
Smartphone	67.09±20.70	68.93±18.33	74.78±16.48	75.00±15.00	78.64±17.50	79.19±14.04	78.40±6.06	74.13±14.57

AX-CPT	52.27±9.30	65.90±6.88	67.95±20.59	74.77±16.80	77.04±26.01	73.40±15.08	74.00±3.57	69.80±5.95

- **Stroke rate (strokes per minutes)**

	1st 200 m	2nd 200 m	3rd 200 m	4th 200m	5th 200m	6th 200m	Average of first 6 200m
Documentary	30.7±2.0	31.6±2.6	31.4±3.1	31.9±2.3	32.8±2.9	31.9±3.2	31.8±2.5
Smartphone	30.8±3.2	31.4±2.8	31.9±2.7	32.3±3.2	32.8±3.3	32.3±3.2	31.9±2.9
AX-CPT	31.1±3.6	32.5±3.6	32.0±2.1	31.7±2.1	32.3±2.6	32.7±2.8	32.0±2.6

- **Index of Coordination (%)**

	1st 200 m	2nd 200 m	3rd 200 m	4th 200m	5th 200m	6th 200m	Average of first 6 200m
Documentary	-8.01±9.21	-7.68±6.32	-7.83±8.20	-8.09±8.55	-7.22±9.05	-8.41±10.50	-7.87±8.63
Smartphone	-9.39±6.64	-8.61±5.83	-8.01±9.23	-6.17±7.61	-7.74±5.52	-7.82±2.72	-7.92±5.79
AX-CPT	-8.53±10.77	-8.90±5.01	-7.36±5.88	-7.03±6.99	-7.58±7.53	-6.36±6.76	-7.35±6.41

- **Propulsive phase (% of stroke cycle)**

	1st 200 m	2nd 200 m	3rd 200 m	4th 200m	5th 200m	6th 200m	Average of first 6 200m
Documentary	40.12±7.2	42.27±5.5	39.24±6.1	38.8±7.7	43.0±9.4	39.7±8.0	40.1±5.5
Smartphone	42.3±7.1	40.6±8.3	45.2±11.4	45.2±7.4	41.2±5.9	40.4±4.3	43.2±5.9
AX-CPT	43.8±2.2	40.7±10.6	42.7±7.3	43.3±8.7	44.1±6.5	46.0±7.0	43.6±5.5

- **Non-propulsive phase (% of stroke cycle)**

	1st 200 m	2nd 200 m	3rd 200 m	4th 200m	5th 200m	6th 200m	Average of first 6 200m
Documentary	58.6±9.8	57.7±6.4	60.8±6.2	61.1±7.7	57.0±9.2	60.3±8.3	59.9±5.1

Smartphone	57.7±5.8	59.4±8.3	54.8±10.8	55.1±6.3	58.8±6.8	60.1±5.7	56.9±5.9
AX-CPT	57.8±8.4	59.3±10.6	57.3±8.0	58.2±8.0	55.3±6.9	54.2±7.1	56.9±7.2

- **Entry phase (% of stroke cycle)**

	1st 200 m	2nd 200 m	3rd 200 m	4th 200m	5th 200m	6th 200m	Average of first 6 200m
Documentary	39.2±10.9	35.9±5.8	38.6±6.2	39.8±9.1	37.8±11.3	39.6±8.0	38.9±4.6
Smartphone	38.0±8.7	38.6±11.1	33.3±15.2	34.7±13.8	39.8±6.9	40.4±7.4	36.2±7.5
AX-CPT	38.3±4.7	39.2±9.0	36.6±6.6	39.4±6.8	35.0±12.7	35.2±9.6	36.7±6.7

- **Pull phase (% of stroke cycle)**

	1st 200 m	2nd 200 m	3rd 200 m	4th 200m	5th 200m	6th 200m	Average of first 6 200m
Documentary	18.2±5.0	20.0±4.7	19.2±2.7	18.8±5.2	19.1±2.9	17.8±3.5	18.4±3.4
Smartphone	18.3±3.0	19.1±64.8	20.8±6.8	21.2±3.1	19.0±3.4	19.0±2.5	20.0±2.9
AX-CPT	17.2±6.8	19.4±1.7	18.9±3.8	19.0±3.1	19.4±2.2	20.4±0.4	19.5±2.0

- **Push phase (% of stroke cycle)**

	1st 200 m	2nd 200 m	3rd 200 m	4th 200m	5th 200m	6th 200m	Average of first 6 200m
Documentary	22.3±9.0	22.3±3.9	20.5±3.9	20.9±3.7	22.61±9.8	22.5±7.0	22.0±3.6
Smartphone	23.1±4.9	21.1±4.2	22.7±7.7	24.0±4.7	24.0±2.5	20.7±3.2	22.9±3.4
AX-CPT	26.3±8.0	21.1±9.1	23.8±3.7	22.3±7.6	25.3±4.9	25.5±7.0	23.4±5.1

- **Exit phase (% of stroke cycle)**

	1st 200 m	2nd 200 m	3rd 200 m	4th 200m	5th 200m	6th 200m	Average of first 6 200m
Documentary	20.0±2.8	21.3±1.9	21.7±3.0	21.1±1.9	19.2±2.1	19.9±1.9	20.6±1.3
Smartphone	18.1±5.7	19.5±4.2	21.5±6.0	20.2±8.2	20.0±1.7	21.4±5.2	20.2±2.7
AX-CPT	20.1±4.8	19.2±3.0	20.9±1.7	18.8±2.0	20.2±5.9	19.0±2.7	19.7±1.1

- **Start of the Breathing action (% of stroke cycle - right)**

	1st 200 m	2nd 200 m	3rd 200 m	4th 200m	5th 200m	6th 200m	Average of first 6 200m	7th 200m (maximal)
Documentary	34.29±56.83	29.00±55.44	56.33±	30.18±	25.05±	30.44±	32.43±55.65	30.12±
Smartphone	-2.66±	6.98±	34,46±	5.52±	36.70±	36.41±	12.20±	14.10±
AX-CPT	27.27±54.45	56.93±	47,15±52.66	37.24±46.34	41.93±55.97	29.01±65.19	22.85±53.46	53.09±

- **Start of the Breathing action (% of stroke cycle - left)**

	1st 200 m	2nd 200 m	3rd 200 m	4th 200m	5th 200m	6th 200m	Average of first 6 200m	7th 200m (maximal)
Documentary	18.00±45.15	21.09±35.87	16.11±27.60	16.10±27.87	19.35±28.19	17.03±27.79	17.64±38.41	16.74±28.91
Smartphone	23.46±	9.76±	11.62±	10.22±	19.06±	15.49±	10.65±	16.26±
AX-CPT	11.92±35.56	17.86±	14.39±28.36	13.65±29.66	15.79±25.91	16.00±21.75	11.44±34.67	9.70±

- **End of the Breathing action (% of stroke cycle - right)**

	1st 200 m	2nd 200 m	3rd 200 m	4th 200m	5th 200m	6th 200m	Average of first 6 200m	7th 200m (maximal)
Documentary	74.31±40.32	70.21±39.50	89.54±	75.00±	70.28±	75.51±	73.89±39.55	76.27±

Smartphone	42.47±	53.43±	73.84±	55.49±	76.09±	73.84±	54.59±	55.54±
AX-CPT	66.38±36.27	95.93±	86.66±36.55	69.94±37.95	81.65±41.39	74.57±51.04	63.41±36.73	95.37±

- **End of the Breathing action (% of stroke cycle - left)**

	1st 200 m	2nd 200 m	3rd 200 m	4th 200m	5th 200m	6th 200m	Average of first 6 200m	7th 200m (maximal)
Documentary	50.57±53.80	61.56±48.05	51.40±35.18	53.42±36.38	56.88±37.64	53.54±36.17	55.20±47.91	53.95±37.23
Smartphone	68.09±	52.25±	50.95±	52.09±	57.56±	54.33±	51.81±	57.05±
AX-CPT	48.55±32.79	51.61±	50.03±36.69	51.29±37.51	51.99±33.70	53.57±29.57	50.43±32.68	50.34±

- **First right Kick (% of stroke cycle - right)**

	1st 200 m	2nd 200 m	3rd 200 m	4th 200m	5th 200m	6th 200m	Average of first 6 200m	7th 200m (maximal)
Documentary	20.46±10.84	21.10±12.35	26.59±	25.59±	26.52±	29.24±	21.91±12.69	30.83±
Smartphone	23.60±31.21	26.52±12.97	22.23±21.90	27.63±11.45	29.95±	27.43±	24.79±17.83	33.23±
AX-CPT	26.10±19.92	21.41±10.88	19.65±16.57	24.86±22.20	27.55±17.92	24.41±19.33	24.60±17.45	26.27±18.50

- **First right Kick (% of stroke cycle - left)**

	1st 200 m	2nd 200 m	3rd 200 m	4th 200m	5th 200m	6th 200m	Average of first 6 200m	7th 200m (maximal)
Documentary	24.16±20.49	20.15±20.64	23.85±18.40	25.68±17.88	21.86±18.19	22.83±17.88	22.54±20.26	20.82±17.18
Smartphone	16.74±18.41	14.97±31.82	14.86±30.10	15.41±24.75	21.38±	20.28±	22.56±25.49	21.34±
AX-CPT	21.90±26.79	16.66±27.86	20.13±28.14	22.93±17.79	21.62±19.36	20.43±23.34	21.01±23.76	19.74±12.28

- **Second right Kick (% of stroke cycle - right)**

	1st 200 m	2nd 200 m	3rd 200 m	4th 200m	5th 200m	6th 200m	Average of first 6 200m	7th 200m (maximal)
Documentary	53.95±12.05	53.84±12.46	61.01±	60.19±	59.75±	61.61±	55.03±12.69	63.94±
Smartphone	56.38±13.94	59.81±12.63	56.45±22.92	63.25±12.65	64.39±	62.03±	54.12±15.19	66.27±
AX-CPT	58.79±11.12	53.23±11.88	52.83±16.39	56.34±11.82	60.15±9.52	53.26±9.47	56.50±10.15	59.49±16.30

- **Second right Kick (% of stroke cycle - left)**

	1st 200 m	2nd 200 m	3rd 200 m	4th 200m	5th 200m	6th 200m	Average of first 6 200m	7th 200m (maximal)
Documentary	45.42±17.30	46.85±16.87	47.21±20.17	48.80±21.31	47.02±19.86	46.94±24.67	42.23±18.00	47.72±18.25
Smartphone	42.98±14.23	47.50±27.22	46.85±26.51	46.18±21.39	50.57±	49.65±	49.59±21.90	49.55±
AX-CPT	51.52±18.34	50.24±26.23	57.09±29.18	58.44±18.59	59.22±21.56	60.22±21.58	58.76±22.03	51.43±12.10

- **Third right Kick (% of stroke cycle - right)**

	1st 200 m	2nd 200 m	3rd 200 m	4th 200m	5th 200m	6th 200m	Average of first 6 200m	7th 200m (maximal)
Documentary	77.44±36.92	74.66±23.39	78.59±	74.68±	77.55±	76.52±	75.50±29.22	94.72±
Smartphone	76.22±53.60	62.20±52.57	52.39±54.01	69.78±39.91	76.28±	65.89±	65.59±39.65	89.09±
AX-CPT	81.26±78.56	78.22±38.52	70.60±65.17	68.29±69.09	77.41±52.55	76.85±61.74	77.83±61.51	81.86±17.16

- **Third right Kick (% of stroke cycle - left)**

	1st 200 m	2nd 200 m	3rd 200 m	4th 200m	5th 200m	6th 200m	Average of first 6 200m	7th 200m (maximal)
Documentary	71.21±40.28	70.46±38.19	73.39±64.03	71.47±62.99	70.02±61.27	67.70±65.41	70.83±41.51	76.96±46.75

Smartphone	69.55±44.49	66.05±38.81	61.10±40.96	69.70±30.81	69.94±	64.51±	69.24±32.08	69.83±
AX-CPT	69.56±72.09	70.17±31.94	68.79±51.23	67.86±48.57	66.21±40.49	68.79±56.44	68.56±50.74	78.52±11.98

- **Duration of first right Kick (% of stroke cycle - right)**

	1st 200 m	2nd 200 m	3rd 200 m	4th 200m	5th 200m	6th 200m	Average of first 6 200m	7th 200m (maximal)
Documentary	33.27±3.22	32.23±4.81	31.68±	30.85±	30.79±	29.99±	32.06±4.59	28.56±
Smartphone	32.75±3.20	33.44±3.62	33.13±2.19	33.40±5.83	31.99±	31.94±	32.38±3.52	31.86±
AX-CPT	32.91±3.05	31.85±4.26	32.32±3.69	31.87±3.32	32.22±4.40	31.44±3.78	32.29±3.69	33.29±7.28

- **Duration of first right Kick (% of stroke cycle - left)**

	1st 200 m	2nd 200 m	3rd 200 m	4th 200m	5th 200m	6th 200m	Average of first 6 200m	7th 200m (maximal)
Documentary	32.34±7.13	32.13±7.00	32.98±7.14	32.90±7.21	33.04±6.50	32.97±7.41	32.12±6.75	33.20±6.19
Smartphone	31.65±3.75	31.89±6.67	31.83±6.18	31.78±6.85	32.50±	32.83±	32.62±5.22	32.43±
AX-CPT	32.04±5.84	33.22±6.15	32.36±6.24	33.18±6.33	32.41±5.77	32.40±5.34	32.36±5.92	32.93±6.01

- **Duration of second right Kick (% of stroke cycle - right)**

	1st 200 m	2nd 200 m	3rd 200 m	4th 200m	5th 200m	6th 200m	Average of first 6 200m	7th 200m (maximal)
Documentary	31.94±1.76	32.38±3.27	34.15±	31.59±	31.79±	32.40±	32.11±2.47	33.99±
Smartphone	32.10±7.73	31.45±4.35	30.57±7.52	31.45±2.67	29.06±	28.93±	32.16±5.27	32.03±
AX-CPT	30.46±9.48	31.48±5.90	31.59±6.80	30.73±7.55	31.86±5.87	31.58±8.40	31.12±7.05	33.11±5.09

- **Duration of second right Kick (% of stroke cycle - left)**

	1st 200 m	2nd 200 m	3rd 200 m	4th 200m	5th 200m	6th 200m	Average of first 6 200m	7th 200m (maximal)
Documentary	33.04±4.87	33.10±5.01	33.49±9.62	33.34±8.18	32.85±8.24	33.11±11.34	33.07±6.45	34.19±7.75
Smartphone	31.82±5.78	33.45±6.27	32.46±5.68	33.84±3.67	34.73±	34.72±	32.25±3.89	35.90±
AX-CPT	31.94±9.15	32.86±3.63	31.76±5.00	32.68±4.89	32.51±4.56	31.54±5.89	31.82±5.04	34.48±5.79

- **Duration of third right Kick (% of stroke cycle - right)**

	1st 200 m	2nd 200 m	3rd 200 m	4th 200m	5th 200m	6th 200m	Average of first 6 200m	7th 200m (maximal)
Documentary	31.07±10.11	31.49±7.80	33.14±	29.73±	30.35±	29.28±	30.19±8.88	36.31±
Smartphone	29.66±19.98	23.33±18.18	19.81±19.39	26.25±11.74	28.06±	24.80±	24.97±15.31	33.10±
AX-CPT	31.11±29.22	30.71±13.79	26.69±24.58	29.02±24.52	28.78±19.67	27.55±23.03	28.32±23.21	33.35±7.49

- **Duration of third right Kick (% of stroke cycle - left)**

	1st 200 m	2nd 200 m	3rd 200 m	4th 200m	5th 200m	6th 200m	Average of first 6 200m	7th 200m (maximal)
Documentary	32.07±21.34	32.55±16.79	27.21±24.76	26.27±24.30	27.12±23.27	25.40±25.27	32.04±20.98	27.75±15.52
Smartphone	31.68±19.86	28.24±24.36	26.45±25.15	32.17±18.26	28.30±	25.10±	31.52±17.48	31.52±
AX-CPT	31.52±31.79	31.51±17.82	29.13±26.28	23.83±21.88	30.18±21.95	30.80±27.58	30.82±25.50	30.04±9.07

- **First left Kick (% of stroke cycle - right)**

	1st 200 m	2nd 200 m	3rd 200 m	4th 200m	5th 200m	6th 200m	Average of first 6 200m	7th 200m (maximal)
Documentary	9.73±	9.28±	9.18±	±	±	±	9.50±	±

Smartphone	13.43±8.96	13.66±10.30	14.33±9.57	14.26±8.05	13.90±	14.21±	14.00±9.41	13.47±
AX-CPT	13.18±	13.24±	13.31±	15.23±	13.88±	14.80±	13.28±	14.50±

- **First left Kick (% of stroke cycle - left)**

	1st 200 m	2nd 200 m	3rd 200 m	4th 200m	5th 200m	6th 200m	Average of first 6 200m	7th 200m (maximal)
Documentary	18.92±	18.72±	19.20±	19.10±	19.01±	18.63±	18.93±	17.82±
Smartphone	17.93±8.62	21.81±18.50	19.61±9.63	17.57±9.24	18.06±	17.36±	19.04±5.21	16.10±
AX-CPT	18.46±	20.81±	22.02±	19.17±	20.78±	22.51±	19.58±	20.99±

- **Second left Kick (% of stroke cycle - right)**

	1st 200 m	2nd 200 m	3rd 200 m	4th 200m	5th 200m	6th 200m	Average of first 6 200m	7th 200m (maximal)
Documentary	39.71±	39.30±	39.08±	±	±	±	39.32±	±
Smartphone	45.21±15.87	45.01±30.76	45.39±16.30	45.86±13.43	43.74±	44.16±	45.41±18.70	43.62±
AX-CPT	46.04±	45.35±	47.14±	49.60±	46.87±	49.85±	46.80±	47.96±

- **Second left Kick (% of stroke cycle - left)**

	1st 200 m	2nd 200 m	3rd 200 m	4th 200m	5th 200m	6th 200m	Average of first 6 200m	7th 200m (maximal)
Documentary	50.06±	51.11±	55.40±	49.41±	55.33±	49.58±	51.06±	53.20±
Smartphone	52.94±8.54	54.54±34.53	54.17±8.29	52.48±8.97	49.23±	53.33±	53.19±12.52	49.55±
AX-CPT	54.98±	56.47±	55.58±	53.28±	56.24±	56.73±	55.58±	54.34±

- **Third left Kick (% of stroke cycle - right)**

	1st 200 m	2nd 200 m	3rd 200 m	4th 200m	5th 200m	6th 200m	Average of first 6 200m	7th 200m (maximal)
Documentary	65.03±	66.71±	68.99±	±	±	±	66.94±	±
Smartphone	75.43±19.33	70.62±57.97	74.87±15.18	77.80±18.03	63.64±	62.45±	71.69±24.11	76.80±
AX-CPT	78.29±	77.47±	76.40±	72.31±	76.89±	71.97±	76.41±	78.44±

- **Third left Kick (% of stroke cycle - left)**

	1st 200 m	2nd 200 m	3rd 200 m	4th 200m	5th 200m	6th 200m	Average of first 6 200m	7th 200m (maximal)
Documentary	77.56±	77.75±	78.27±	78.25±	78.28±	78.33±	78.07±	75.42±
Smartphone	81.17±27.34	66.06±74.98	81.88±30.89	79.73±26.68	78.68±	78.61±	75.02±34.66	79.08±
AX-CPT	86.15±	87.82±	84.58±	66.86±	82.22±	69.70±	84.38±	76.13±

- **Duration of first left Kick (% of stroke cycle - right)**

	1st 200 m	2nd 200 m	3rd 200 m	4th 200m	5th 200m	6th 200m	Average of first 6 200m	7th 200m (maximal)
Documentary	29.34±	29.36±	29.10±	±	±	±	29.29±	±
Smartphone	29.13±7.43	27.63±8.46	29.11±7.46	29.62±7.62	29.42±	29.67±	28.76±7.81	30.04±
AX-CPT	28.63±	28.27±	29.32±	33.09±	29.61±	31.60±	29.33±	32.16±

- **Duration of first left Kick (% of stroke cycle - left)**

	1st 200 m	2nd 200 m	3rd 200 m	4th 200m	5th 200m	6th 200m	Average of first 6 200m	7th 200m (maximal)
Documentary	32.94±	32.98±	33.51±	33.27±	33.49±	33.44±	33.27±	33.19±

Smartphone	34.36±2.36	32.98±6.07	34.35±3.85	34.74±3.42	33.77±	33.95±	33.99±3.25	34.30±
AX-CPT	33.17±	35.05±	33.10±	32.95±	33.48±	34.14±	33.13±	33.63±

- **Duration of second left Kick (% of stroke cycle - right)**

	1st 200 m	2nd 200 m	3rd 200 m	4th 200m	5th 200m	6th 200m	Average of first 6 200m	7th 200m (maximal)
Documentary	37.12±	36.84±	36.74±	±	±	±	36.77±	±
Smartphone	35.94±1.55	36.00±21.16	36.28±1.58	36.35±1.02	36.64±	36.33±	35.41±4.30	35.66±
AX-CPT	36.11±	36.47±	36.56±	35.98±	36.99±	36.15±	36.57±	36.80±

- **Duration of second left Kick (% of stroke cycle - left)**

	1st 200 m	2nd 200 m	3rd 200 m	4th 200m	5th 200m	6th 200m	Average of first 6 200m	7th 200m (maximal)
Documentary	27.73±	28.36±	29.51±	27.98±	29.59±	27.95±	28.01±	31.11±
Smartphone	28.72±1.22	27.67±18.82	28.78±4.00	29.13±3.44	29.41±	29.71±	28.19±5.24	30.07±
AX-CPT	28.70±	28.40±	29.62±	30.33±	29.10±	29.50±	29.59±	29.53±

- **Duration of second left Kick (% of stroke cycle - right)**

	1st 200 m	2nd 200 m	3rd 200 m	4th 200m	5th 200m	6th 200m	Average of first 6 200m	7th 200m (maximal)
Documentary	31.09±	31.58±	32.72±	±	±	±	31.80±	±
Smartphone	31.49±10.68	27.01±29.36	33.01±10.15	31.63±9.95	33.59±	33.48±	30.24±13.11	33.86±
AX-CPT	32.68±	34.97±	32.27±	27.20±	32.33±	28.49±	32.57±	30.72±

- **Duration of third left Kick (% of stroke cycle - left)**

	1st 200 m	2nd 200 m	3rd 200 m	4th 200m	5th 200m	6th 200m	Average of first 6 200m	7th 200m (maximal)
Documentary	36.55±	36.83±	36.66±	35.65±	36.61±	36.78±	36.78±	33.60±
Smartphone	35.98±6.36	29.52±31.19	36.67±11.67	36.16±7.60	35.91±	36.11±	33.51±11.79	35.38±
AX-CPT	36.89±	36.19±	36.37±	29.74±	35.40±	30.32±	36.37±	32.70±

- **Left hand entry (% of stroke cycle - right)**

	1st 200 m	2nd 200 m	3rd 200 m	4th 200m	5th 200m	6th 200m	Average of first 6 200m	7th 200m (maximal)
Documentary	53.37±10.68	53.30±9.38	52.94±	51.97±	53.53±	53.18±	53.56±9.44	52.88±
Smartphone	53.35±9.48	52.75±9.40	52.97±9.07	55.63±21.19	50.33±9.82	50.67±10.36	53.43±7.23	49.73±12.39
AX-CPT	51.15±9.17	53.50±10.27	51.37±9.01	50.04±10.16	51.76±9.10	51.14±8.57	51.51±9.20	49.32±7.81

- **Right hand entry (% of stroke cycle - left)**

	1st 200 m	2nd 200 m	3rd 200 m	4th 200m	5th 200m	6th 200m	Average of first 6 200m	7th 200m (maximal)
Documentary	45.47±32.94	46.55±9.33	47.06±	48.12±	46.34±	46.69±	44.77±19.84	47.23±
Smartphone	45.23±8.38	47.09±9.39	46.85±37.19	44.18±20.40	49.51±9.45	48.76±9.84	46.22±13.97	49.87±11.61
AX-CPT	48.72±9.10	46.31±9.86	48.47±8.34	49.99±9.68	48.13±9.81	48.63±8.15	48.38±15.68	50.22±8.15

- **Kick rate - right (kicks per second)**

	1st 200 m	2nd 200 m	3rd 200 m	4th 200m	5th 200m	6th 200m	Average of first 6 200m	7th 200m (maximal)
Documentary	1.55±0.18	1.58±0.20	1.60±0.34	1.62±0.31	1.62±0.30	1.64±0.39	1.61±0.25	1.77±0.31

Smartphone	1.53±0.21	1.49±0.19	1.48±0.15	1.58±0.13	1.56±0.15	1.56±0.15	1.51±0.13	1.75±0.20
AX-CPT	1.48±0.52	1.54±0.16	1.49±0.49	1.64±0.52	1.63±0.39	1.63±0.40	1.61±0.41	1.74±0.37

- **Kick rate – left (kicks per second)**

	1st 200 m	2nd 200 m	3rd 200 m	4th 200m	5th 200m	6th 200m	Average of first 6 200m	7th 200m (maximal)
Documentary	1.57±	1.60±	1.62±	1.62±	1.65±	1.66±	1.62±	1.74±
Smartphone	1.53±0.09	1.48±0.57	1.57±0.14	1.59±0.15	1.65±	1.66±	1.50±0.12	1.72±
AX-CPT	1.49±	1.55±	1.48±	1.56±	1.52±	1.60±	1.50±	1.73±

- **Kick time - right (seconds)**

	1st 200 m	2nd 200 m	3rd 200 m	4th 200m	5th 200m	6th 200m	Average of first 6 200m	7th 200m (maximal)
Documentary	0.68±0.20	0.64±0.16	0.63±0.35	0.62±0.32	0.63±0.32	0.63±0.42	0.64±0.23	0.56±0.23
Smartphone	0.66±0.24	0.70±0.14	0.72±0.14	0.66±0.06	0.67±0.10	0.68±0.15	0.68±0.12	0.59±0.23
AX-CPT	0.66±0.31	0.65±0.10	0.63±0.18	0.62±0.10	0.62±0.10	0.63±0.13	0.63±0.16	0.58±0.06

- **Kick time - left (seconds)**

	1st 200 m	2nd 200 m	3rd 200 m	4th 200m	5th 200m	6th 200m	Average of first 6 200m	7th 200m (maximal)
Documentary	0.64±	0.62±	0.62±	0.61±	0.60±	0.61±	0.62±	0.58±
Smartphone	0.65±0.05	0.68±0.06	0.65±0.05	0.63±0.06	0.61±	0.60±	0.68±0.05	0.58±
AX-CPT	0.63±	0.65±	0.62±	0.56±	0.60±	0.59±	0.62±	0.58±

- **Stroke time - right (seconds)**

	1st 200 m	2nd 200 m	3rd 200 m	4th 200m	5th 200m	6th 200m	Average of first 6 200m	7th 200m (maximal)
Documentary	1.92±0.13	1.88±0.19	1.85±0.15	1.80±0.15	1.82±0.16	1.79±20	1.87±0.19	1.66±0.20
Smartphone	1.95±0.07	1.89±0.18	1.86±0.18	1.84±0.24	1.82±0.16	1.80±0.14	1.86±0.20	1.72±0.16
AX-CPT	1.93±0.09	1.85±0.15	1.86±0.20	1.83±0.17	1.84±0.19	1.83±0.21	1.86±0.17	1.68±0.19

- **Stroke time - left (seconds)**

	1st 200 m	2nd 200 m	3rd 200 m	4th 200m	5th 200m	6th 200m	Average of first 6 200m	7th 200m (maximal)
Documentary	1.92±0.11	1.88±0.16	1.86±0.14	1.84±0.13	1.82±0.09	1.81±0.14	1.85±0.17	1.69±0.17
Smartphone	1.95±0.06	1.89±0.17	1.86±0.18	1.84±0.22	1.82±0.17	1.80±0.15	1.86±0.19	1.73±0.16
AX-CPT	1.92±0.10	1.85±0.14	1.85±0.18	1.83±0.16	1.82±0.17	1.81±0.18	1.85±0.15	1.69±0.19

- **Number of right strokes for each length**

	1st 200 m	2nd 200 m	3rd 200 m	4th 200m	5th 200m	6th 200m	Average of first 6 200m	7th 200m (maximal)
Documentary	14.25±3.00	14.58±2.92	14.66±	14.08±	13.83±	14.08±	14.69±2.81	14.16±
Smartphone	15.16±4.83	15.00±3.42	15.00±3.58	15.33±3.33	15.25±3.62	15.33±3.79	15.30±3.38	16.16±3.67
AX-CPT	14.50±3.75	14.50±3.42	15.00±3.33	14.91±3.29	15.00±3.33	15.50±3.25	14.83±3.33	16.75±2.50

- **Number of left strokes for each length**

	1st 200 m	2nd 200 m	3rd 200 m	4th 200m	5th 200m	6th 200m	Average of first 6 200m	7th 200m (maximal)
Documentary	13.33±3.92	14.16±4.25	14.16±3.92	14.16±4.29	14.41±3.67	14.41±4.00	14.27±3.97	14.91±3.79
Smartphone	14.83±4.96	14.33±4.25	14.66±4.58	14.83±3.83	14.41±3.96	14.75±3.58	14.76±4.07	15.50±3.58
AX-CPT	14.00±4.75	13.91±4.00	14.16±4.17	14.50±3.63	14.50±4.00	14.83±4.00	14.27±4.12	16.25±3.42

- **Right stroke length (metres)**

	1st 200 m	2nd 200 m	3rd 200 m	4th 200m	5th 200m	6th 200m	Average of first 6 200m	7th 200m (maximal)
Documentary	2.13±0.42	2.09±0.34	2.08±0.36	2.15±0.38	2.17±0.34	2.15±0.34	2.07±0.36	2.12±0.40
Smartphone	2.05±0.59	2.02±0.45	2.03±0.45	1.95±0.40	2.01±0.46	1.99±0.45	2.02±0.42	1.91±0.45
AX-CPT	2.13±0.48	2.11±0.42	2.03±0.41	2.08±0.45	2.03±0.44	1.97±0.40	2.03±0.42	1.86±0.27

- **Left stroke length (metres)**

	1st 200 m	2nd 200 m	3rd 200 m	4th 200m	5th 200m	6th 200m	Average of first 6 200m	7th 200m (maximal)
Documentary	2.10±0.45	2.10±0.40	2.06±0.45	2.07±0.48	2.06±0.47	2.05±0.47	2.04±0.42	1.97±0.46
Smartphone	2.06±0.62	2.04±0.47	2.05±0.47	1.97±0.42	2.03±0.44	2.02±0.46	2.00±0.44	1.92±0.47
AX-CPT	2.13±0.52	2.13±0.42	2.03±0.41	2.10±0.47	2.06±0.48	1.97±0.44	2.09±0.44	1.86±0.30

- **Stroke index (right)**

	1st 200 m	2nd 200 m	3rd 200 m	4th 200m	5th 200m	6th 200m	Average of first 6 200m	7th 200m (maximal)
Documentary	2.39±0.94	2.33±0.92	2.31±0.90	2.53±0.96	2.54±0.93	2.54±0.94	2.30±0.93	2.65±0.95

Smartphone	2.13±1.30	2.27±1.07	2.30±1.07	2.16±1.01	2.24±1.05	2.17±0.99	2.20±1.03	2.19±0.95
AX-CPT	2.41±1.12	2.36±1.07	2.29±1.05	2.36±1.01	2.30±1.02	2.24±0.99	2.33±1.05	2.05±0.77

- **Stroke index (left)**

	1st 200 m	2nd 200 m	3rd 200 m	4th 200m	5th 200m	6th 200m	Average of first 6 200m	7th 200m (maximal)
Documentary	2.54±1.12	2.39±1.20	2.42±1.18	2.36±1.11	2.26±1.19	2.30±1.22	2.36±1.14	2.39±1.06
Smartphone	2.20±1.38	2.38±1.21	2.35±1.24	2.24±1.09	2.37±1.13	2.26±1.02	2.28±1.16	2.29±1.00
AX-CPT	2.50±1.32	2.46±1.18	2.40±1.23	2.43±1.11	2.36±1.14	2.34±1.10	2.42±1.19	2.11±0.90

- **Number of right kicks for each length**

	1st 200 m	2nd 200 m	3rd 200 m	4th 200m	5th 200m	6th 200m	Average of first 6 200m	7th 200m (maximal)
Documentary	39.66±9.25	42.83±8.17	40.58±9.21	40.58±8.42	40.33±8.71	40.50±9.87	42.33±9.96	43.00±7.83
Smartphone	39.75±11.50	42.75±14.71	42.58±14.08	44.58±14.04	38.50±	40.00±	42.00±11.57	44.83±
AX-CPT	35.00±16.67	41.08±13.17	39.50±12.83	40.00±7.58	41.66±11.67	39.00±13.33	37.52±12.21	50.16±7.87

- **Number of left kicks for each length**

	1st 200 m	2nd 200 m	3rd 200 m	4th 200m	5th 200m	6th 200m	Average of first 6 200m	7th 200m (maximal)
Documentary	37.66±	42.33±	40.00±	41.16±	40.66±	38.83±	39.02±	45.00±
Smartphone	45.83±12.33	43.50±24.87	47.50±10.83	47.33±11.71	45.00±	45.33±	45.15±10.77	47.50±
AX-CPT	49.00±	44.41±	49.00±	43.75±	46.83±	51.25±	49.53±	55.16±

- **Total number of breathing actions for each length**

	1st 200 m	2nd 200 m	3rd 200 m	4th 200m	5th 200m	6th 200m	Average of first 6 200m	7th 200m (maximal)
Documentary	12.66±2.17	13.50±2.25	13.75±2.00	13.41±3.04	13.58±2.38	13.66±3.00	13.72±2.39	14.08±3.21
Smartphone	12.75±2.10	13.83±2.50	13.50±2.40	14.33±2.20	14.33±2.40	14.33±2.30	14.06±2.20	15.33±3.00
AX-CPT	12.83±2.50	13.16±2.30	13.66±3.08	13.75±2.67	13.91±2.42	14.08±2.62	13.55±2.30	14.83±3.00
