# Supplementary material for: Does Smartphone Use Affect a Subsequent Swimming Training Session? Preliminary Results in Amateur Triathletes
Source: Sensors (Basel). 2023 Jun 23;23(13):5837. doi: 10.3390/s23135837 (PMC10347285; doi:10.3390/s23135837)
Supplement: Supplementary file 1 [file sensors-23-05837-s001.zip › S2-Statistical Analysis.pdf]

Statistical Analysis

- **Mental Fatigue VAS**

Between condition

Pre	Post task	Post session
$H_{(19,2)}$=1.038<br>p=.595	$H_{(19,2)}$=8.969<br>p=.011*	$H_{(19,2)}$=0.704<br>p=.703

Post-hoc

	Documentary - Smartphone	Documentary – AX-CPT	Smartphone - AXCPT
Post task	$U_{(13)}$=12.00<br>p=.128<br>r=.045	$U_{(13)}$=2.000<br>p=.002*<br>r=.092	$U_{(13)}$=12.500<br>p=.128<br>r=-.043

Within condition

Documentary	Smartphone	AX-CPT
$X^2_{(7,2)}$= 4.692<br>p=.096<br>W=.335	$X^2_{(7,2)}$= 1.143<br>p=.565<br>W=.082	$X^2_{(7,2)}$= 3.714<br>p=.156<br>W=.265

- **Motivation VAS**

Between condition

Pre	Post task	Post session
$H_{(19,2)}$=0.365<br>p=.833	$H_{(19,2)}$=0.147<br>p=.929	$H_{(19,2)}$=0.677<br>p=.713

Within condition

Documentary	Smartphone	AX-CPT
$X^2_{(7,2)}$= 6.333<br>p=.042*<br>W=.452	$X^2_{(7,2)}$= 7.913<br>p=.019*<br>W=.565	$X^2_{(7,2)}$= 2.333<br>p=.311<br>W=.167

Post-hoc

	Pre – Post task	Pre – Post session	Post task – Post session
Documentary	$U_{(6)}$=-2.201<br>p=.028*<br>r=.974	$U_{(6)}$=-0.526<br>p=.599<br>r=.070	$U_{(6)}$=-1.572<br>p=.116<br>r=.108
Smartphone	$U_{(6)}$=-1.782<br>p=.075<br>r=.729	$U_{(6)}$=-1.753<br>p=.080<br>r=.981	$U_{(6)}$=-2.201<br>p=.028*<br>r=.637

- **Short-Stroop task – Reaction time**

Between condition

Pre	Post task	Post session
$H_{(19,2)}$=1.744<br>p=.418	$H_{(19,2)}$=0.364<br>p=.834	$H_{(19,2)}$=3.258<br>p=.196

Within condition

Documentary	Smartphone	AX-CPT
$X^2_{(7,2)}$= 5.492<br>p=.066<br>W=.388	$X^2_{(7,2)}$= 8.857<br>p=.012*<br>W=.633	$X^2_{(7,2)}$= 4.000<br>p=.135<br>W=.333

Post-hoc

	Pre – Post task	Pre – Post session	Post task – Post session
Smartphone	$U_{(6)}$=-1.014<br>p=.310<br>r=.697	$U_{(6)}$=-2.366<br>p=.018*<br>r=.773	$U_{(6)}$=-2.197<br>p=.028*<br>r=.881

- **Short-Stroop task – Number of correct answers**

Between condition

Pre	Post task	Post session
$H_{(19,2)}$=1.920<br>p=.383	$H_{(19,2)}$=1.562<br>p=.458	$H_{(19,2)}$=0.926<br>p=.629

Within condition

Documentary	Smartphone	AX-CPT
$X^2_{(7,2)}$= 2.600<br>p=.273<br>W=.186	$X^2_{(7,2)}$= 1.500<br>p=.472<br>W=.107	$X^2_{(7,2)}$= 5.200<br>p=.074<br>W=.433

- **Short-Stroop task – Reaction time of correct answers**

Between condition

Pre	Post task	Post session
$H_{(19,2)}$=1.597<br>p=.450	$H_{(19,2)}$=0.356<br>p=.837	$H_{(19,2)}$=3.325<br>p=.190

Within condition

Documentary	Smartphone	AX-CPT
$X^2_{(7,2)}$= 5.429<br>p=.066<br>W=.388	$X^2_{(7,2)}$= 8.857<br>p=.012*<br>W=.633	$X^2_{(7,2)}$= 4.000<br>p=.135<br>W=.333

Post-hoc

	Pre – Post task	Pre – Post session	Post task – Post session
Smartphone	$U_{(6)}$=-1.183<br>p=.237<br>r=.767	$U_{(6)}$=-2.366<br>p=.018*<br>r=.767	$U_{(6)}$=-2.197<br>p=.028*<br>r=.864

- **AX-CPT**

Section of total task time analysed	Time of answer	Number of errors	Reaction time of correct answer
Each third	$X^2_{(7,2)}$=5.429<br>P=.066<br>W=.388	$X^2_{(7,2)}$=1.455<br>P=.483<br>W=.104	$X^2_{(7,2)}$=2.000<br>P=.368<br>W=.143
First and last third	$Z_{(6)}$=-1.521<br>p=.128<br>r=.930	$Z_{(6)}$=-0.106<br>p=.916<br>r=.903	$Z_{(6)}$=-1.014<br>p=.310<br>r=.430

- **ITAMS**

Documentary

Anger	Confusion	Depression	Fatigue	Tension	Vigor
$H_{(19,2)}$=.359<br>p=.836	$H_{(19,2)}$=.386<br>p=.824	$H_{(19,2)}$=1.203<br>p=.548	$H_{(19,2)}$=.689<br>p=.709	$H_{(19,2)}$=.763<br>p=.683	$H_{(19,2)}$=6.469<br>p=.039*

Post-hoc

	Pre – Post task	Pre – Post session	Post task – Post session
Vigor	$U_{(14)}$=14.500<br>p=.113	$U_{(14)}$=17.500<br>p=.216	$U_{(14)}$=6.00<br>p=.017*

Smartphone

Anger	Confusion	Depression	Fatigue	Tension	Vigor
$H_{(19,2)}$=.264<br>p=.876	$H_{(19,2)}$=1.557<br>p=.459	$H_{(19,2)}$=.437<br>p=.804	$H_{(19,2)}$=1.096<br>p=.578	$H_{(19,2)}$=.762<br>p=.683	$H_{(19,2)}$=2.395<br>p=.302

Documentary

Anger	Confusion	Depression	Fatigue	Tension	Vigor
$H_{(19,2)}$=.565<br>p=.754	$H_{(19,2)}$=3.187<br>p=.203	$H_{(19,2)}$=.299<br>p=.861	$H_{(19,2)}$=2.445<br>p=.295	$H_{(19,2)}$=.773<br>p=.679	$H_{(19,2)}$=1.687<br>p=.430

- **Time performance**

Between conditions

1st 200 m	2nd 200 m	3rd 200 m	4th 200m	5th 200m	6th 200m	Average of first 6 200m	7th 200m (maximal)
$H_{(12,2)}$=0.346 p=.841	$H_{(12,2)}$=0.269 p=.874	$H_{(12,2)}$=1.038 p=.595	$H_{(12,2)}$=0.500 p=.779	$H_{(12,2)}$=0.154 p=.926	$H_{(12,2)}$=0.269 p=.874	$H_{(12,2)}$=0.154 p=.926	$H_{(12,2)}$=0.154 p=.926

Within condition

Documentary	Smartphone	AX-CPT
$X^2_{(4,5)}$= 2.286 p=.808 W=.114	$X^2_{(4,5)}$= 13.000 p=.023* W=.650	$X^2_{(4,5)}$= 9.429 p=.093 W=.471

Post-hoc

	1st vs 2nd	1st vs 3rd	1st vs 4th	1st vs 5th	1st vs 6th	2nd vs 3rd	2nd vs 4th	2nd vs 5th
Smartphone	$Z_{(4)}$=-1.461 p=.144 r=.997	$Z_{(4)}$=-1.826 p=.068 r=.997	$Z_{(4)}$=-1.826 p=.068 r=.998	$Z_{(4)}$=-1.461 p=.144 r=.994	$Z_{(4)}$=-1.826 p=.068 r=.995	$Z_{(4)}$=-1.905 p=.273 r=.996	$Z_{(4)}$=-1.461 p=.144 r=.998	$Z_{(4)}$=0.000 p=1.000 r=.997

	2nd vs 6th	3rd vs 4th	3rd vs 5th	3rd vs 6th	4th vs 5th	4th vs 6th	5th vs 6th
Smartphone	$Z_{(4)}$=-1.826 p=.068 r=.997	$Z_{(4)}$=0.000 p=1.000 r=1.000	$Z_{(4)}$=-0.730 p=.465 r=.999	$Z_{(4)}$=-1.826 p=.068 r=.999	$Z_{(4)}$=-0.365 p=.715 r=.999	$Z_{(4)}$=-1.826 p=.068 r=.999	$Z_{(4)}$=-1.826 p=.068 r=1.000

- **Pacing all-out**

Between conditions

1st lap	2nd lap	3rd lap	4th lap	5th lap	6th lap
$H_{(12,2)}$=0.731 p=.694	$H_{(12,2)}$=1.038 p=.595	$H_{(12,2)}$=0.115 p=.944	$H_{(12,2)}$=0.962 p=.618	$H_{(12,2)}$=0.346 p=.841	$H_{(12,2)}$=0.154 p=.926

Within condition

Documentary	Smartphone	AX-CPT
$X^2_{(4,5)}$= 10.143 p=.071 W=.507	$X^2_{(4,5)}$= 13.714 p=.018* W=.686	$X^2_{(4,5)}$= 16.714 p=.005* W=.836

Post-hoc

	1st vs 2nd	1st vs 3rd	1st vs 4th	1st vs 5th	1st vs 6th	2nd vs 3rd	2nd vs 4th	2nd vs 5th	2nd vs 6th
Smartphone	$Z_{(4)}$=-1.826 p=.068 r=.806	$Z_{(4)}$=-1.826 p=.068 r=-.886	$Z_{(4)}$=-1.826 p=.068 r=-.597	$Z_{(4)}$=-1.826 p=.068 r=-.855	$Z_{(4)}$=-1.095 p=.273 r=-.768	$Z_{(4)}$=-0.730 p=.465 r=-.977	$Z_{(4)}$=-1.826 p=.068 r=-.482	$Z_{(4)}$=-0.730 p=.465 r=-.427	$Z_{(4)}$=-0.000 p=1.000 r=-.886
AX-CPT	$Z_{(4)}$=-1.826 p=.068 r=.732	$Z_{(4)}$=-1.826 p=.068 r=.840	$Z_{(4)}$=-1.826 p=.068 r=-.545	$Z_{(4)}$=-1.826 p=.068 r=-.837	$Z_{(4)}$=-1.461 p=.144 r=-.891	$Z_{(4)}$=-1.826 p=.068 r=.967	$Z_{(4)}$=-1.826 p=.068 r=.136	$Z_{(4)}$=-1.826 p=.068 r=-.921	$Z_{(4)}$=-0.365 p=.715 r=-.871

	3rd vs 4th	3rd vs 5th	3rd vs 6th	4th vs 5th	4th vs 6th	5th vs 6th
Smartphone	$Z_{(4)}$=-1.826 p=.068 r=.636	$Z_{(4)}$=-1.461 p=.144 r=.597	$Z_{(4)}$=-1.826 p=.068 r=.820	$Z_{(4)}$=-1.095 p=.273	$Z_{(4)}$=-1.826 p=.068 r=.094	$Z_{(4)}$=-1.461 p=.144

				r=.729		r=.333
AX-CPT	$Z_{(4)}$=-1.826 p=.068 r=-.003	$Z_{(4)}$=-1.095 p=.273 r=-.988	$Z_{(4)}$=-0.730 p=.465 r=-.861	$Z_{(4)}$=-0.365 p=.715 r=.011	$Z_{(4)}$=-1.826 p=.068 r=.327	$Z_{(4)}$=-1.826 p=.068 r=.791

- **RPE**

Between conditions

1st 200 m	2nd 200 m	3rd 200 m	4th 200m	5th 200m	6th 200m	7th 200m (maximal)	Average of first 6 200m
$H_{(12,2)}$=0.432 p=.806	$H_{(12,2)}$=2.953 p=.228	$H_{(12,2)}$=1.285 p=.526	$H_{(12,2)}$=1.953 p=.377	$H_{(12,2)}$=4.465 p=.107	$H_{(12,2)}$=3.537 p=.171	$H_{(12,2)}$=2.999 p=.223	$H_{(12,2)}$=2.615 p=.271

Within condition

Documentary	Smartphone	AX-CPT
$X^2_{(4,5)}$= 22.912 p=.001* W=.955	$X^2_{(4,5)}$= 22.244 p=.001* W=.927	$X^2_{(4,5)}$= 23.370 p=.001* W=.974

Post-hoc

	1st vs 2nd	1st vs 3rd	1st vs 4th	1st vs 5th	1st vs 6th	1st vs 7th
Documentary	$Z_{(4)}$=-2.000 p=.046* r=1.000	$Z_{(4)}$=-1.890 p=.059 r=.944	$Z_{(4)}$=-1.890 p=.059 r=.944	$Z_{(4)}$=-1.826 p=.068 r=.764	$Z_{(4)}$=-1.826 p=.068 r=.697	$Z_{(4)}$=-1.841 p=.066 r=.793
Smartphone	$Z_{(4)}$=-1.342 p=.180 r=.967	$Z_{(4)}$=-1.633 p=.102 r=.944	$Z_{(4)}$=-1.841 p=.066 r=.944	$Z_{(4)}$=-1.633 p=.102 r=.697	$Z_{(4)}$=-1.826 p=.068 r=.369	$Z_{(4)}$=-1.826 p=.068 r=.922
AX-CPT	$Z_{(4)}$=-1.841	$Z_{(4)}$=-1.841	$Z_{(4)}$=-1.826	$Z_{(4)}$=-1.826	$Z_{(4)}$=-1.841	$Z_{(4)}$=-1.841

	p=.066<br>r=.945	p=.066<br>r=.927	p=.068<br>r=.486	p=.068<br>r=.346	p=.066<br>r=.421	p=.066<br>r=.324

- **Heart rate as %HR_{max}**

Between conditions

1st 200 m	2nd 200 m	3rd 200 m	4th 200m	5th 200m	6th 200m	7th 200m (maximal)	Average of first 6 200m
$H_{(12,2)}$=5.115<br>p=.077	$H_{(12,2)}$=4.269<br>p=.118	$H_{(12,2)}$=1.654<br>p=.437	$H_{(12,2)}$=0.500<br>p=.779	$H_{(12,2)}$=0.203<br>p=.904	$H_{(12,2)}$=0.500<br>p=.779	$H_{(12,2)}$=5.683<br>p=.058	$H_{(12,2)}$=2.577<br>p=.276

Within condition

Documentary	Smartphone	AX-CPT
$X^2_{(4,6)}$= 14.905<br>p=.021*<br>W=.621	$X^2_{(4,5)}$= 14.149<br>p=.028*<br>W=.590	$X^2_{(4,5)}$= 19.184<br>p=.004*<br>W=.799

Post-hoc

	1st vs 2nd	1st vs 3rd	1st vs 4th	1st vs 5th	1st vs 6th	1st vs 7th
Documentary	$Z_{(4)}$=-1.461<br>p=.144<br>r=.878	$Z_{(4)}$=-1.826<br>p=.068<br>r=.878	$Z_{(4)}$=-1.826<br>p=.068<br>r=.882	$Z_{(4)}$=-1.826<br>p=.068<br>r=.917	$Z_{(4)}$=-1.826<br>p=.068<br>r=.872	$Z_{(4)}$=-1.826<br>p=.068<br>r=.917
Smartphone	$Z_{(4)}$=-1.604<br>p=.109<br>r=.902	$Z_{(4)}$=-1.826<br>p=.068<br>r=.706	$Z_{(4)}$=-1.826<br>p=.068<br>r=.718	$Z_{(4)}$=-1.826<br>p=.068<br>r=.388	$Z_{(4)}$=-1.826<br>p=.068<br>r=.420	$Z_{(4)}$=-1.826<br>p=.068<br>r=.900
AX-CPT	$Z_{(4)}$=-1.826<br>p=.068<br>r=-.834	$Z_{(4)}$=-1.826<br>p=.068<br>r=-.960	$Z_{(4)}$=-1.826<br>p=.068<br>r=-.910	$Z_{(4)}$=-1.826<br>p=.068<br>r=-.975	$Z_{(4)}$=-1.826<br>p=.068<br>r=-.934	$Z_{(4)}$=-1.826<br>p=.068<br>r=.271

- **Stroke rate**

Between conditions

1st 200 m	2nd 200 m	3rd 200 m	4th 200m	5th 200m	6th 200m	Average of first 6 200m
$H_{(12,2)}$=0.269 p=.874	$H_{(12,2)}$=1.385 p=.500	$H_{(12,2)}$=0.500 p=.779	$H_{(12,2)}$=0.154 p=.926	$H_{(12,2)}$=0.038 p=.981	$H_{(12,2)}$=1.885 p=.390	$H_{(12,2)}$=0.550 p=.760

Within condition

Documentary	Smartphone	AX-CPT
$X^2_{(4,6)}$= 10.468 p=.063 W=.523	$X^2_{(4,5)}$= 7.143 p=.210 W=.357	$X^2_{(4,5)}$=8.143 p=.149 W=.407

- **Index of Coordination**

Between conditions

1st 200 m	2nd 200 m	3rd 200 m	4th 200m	5th 200m	6th 200m	Average of first 6 200m
$H_{(12,2)}$=0.115 p=.944	$H_{(12,2)}$=1.500 p=.472	$H_{(12,2)}$=0.038 p=.981	$H_{(12,2)}$=0.500 p=.779	$H_{(12,2)}$=0.115 p=.944	$H_{(12,2)}$=0.615 p=.735	$H_{(12,2)}$=0.115 p=.944

Within condition

Documentary	Smartphone	AX-CPT
$X^2_{(4,6)}$=3.714 p=.591 W=.186	$X^2_{(4,5)}$= 8.571 p=.127 W=.429	$X^2_{(4,5)}$=9.571 p=.088 W=.479

- **Propulsive phase**

Between conditions

1st 200 m	2nd 200 m	3rd 200 m	4th 200m	5th 200m	6th 200m	Average of first 6 200m
$H_{(12,2)}$=1.423 p=.491	$H_{(12,2)}$=0.154 p=.926	$H_{(12,2)}$=1.654 p=.437	$H_{(12,2)}$=2.000 p=.368	$H_{(12,2)}$=1.038 p=.595	$H_{(12,2)}$=3.577 p=.167	$H_{(12,2)}$=0.808 p=.668

Within condition

Documentary	Smartphone	AX-CPT
$X^2_{(4,6)}$= 4.286<br>p=.509<br>W=.214	$X^2_{(4,5)}$= 4.143<br>p=.529<br>W=.207	$X^2_{(4,5)}$=7.571<br>p=.181<br>W=.379

- **Non-propulsive phase**

Between conditions

1st 200 m	2nd 200 m	3rd 200 m	4th 200m	5th 200m	6th 200m	Average of first 6 200m
$H_{(12,2)}$=0.500<br>p=.779	$H_{(12,2)}$=0.346<br>p=.841	$H_{(12,2)}$=1.192<br>p=.551	$H_{(12,2)}$=1.038<br>p=.595	$H_{(12,2)}$=1.038<br>p=.595	$H_{(12,2)}$=3.500<br>p=.174	$H_{(12,2)}$=0.500<br>p=.779

Within condition

Documentary	Smartphone	AX-CPT
$X^2_{(4,6)}$= 6.714<br>p=.243<br>W=.336	$X^2_{(4,5)}$= 4.000<br>p=.549<br>W=.200	$X^2_{(4,5)}$=10.143<br>p=.071<br>W=.507

- **Entry phase**

Between conditions

1st 200 m	2nd 200 m	3rd 200 m	4th 200m	5th 200m	6th 200m	Average of first 6 200m
$H_{(12,2)}$=0.115<br>p=.944	$H_{(12,2)}$=1.423<br>p=.491	$H_{(12,2)}$=1.846<br>p=.397	$H_{(12,2)}$=1.038<br>p=.595	$H_{(12,2)}$=0.962<br>p=.618	$H_{(12,2)}$=1.192<br>p=.551	$H_{(12,2)}$=0.269<br>p=.874

Within condition

Documentary	Smartphone	AX-CPT
$X^2_{(4,6)}$= 4.143<br>p=.529<br>W=.207	$X^2_{(4,5)}$= 6.714<br>p=.243<br>W=.336	$X^2_{(4,5)}$=2.857<br>p=.722<br>W=.143

- **Pull phase**

Between conditions

1st 200 m	2nd 200 m	3rd 200 m	4th 200m	5th 200m	6th 200m	Average of first 6 200m

$H_{(12,2)}$=0.269 p=.874	$H_{(12,2)}$=0.615 p=.735	$H_{(12,2)}$=0.462 p=.794	$H_{(12,2)}$=1.654 p=.437	$H_{(12,2)}$=0.154 p=.926	$H_{(12,2)}$=3.500 p=.174	$H_{(12,2)}$=0.269 p=.874

Within condition

Documentary	Smartphone	AX-CPT
$X^2_{(4,6)}$= 9.286 p=.098 W=.464	$X^2_{(4,5)}$= 6.000 p=.306 W=.300	$X^2_{(4,5)}$=5.000 p=.416 W=.250

- **Push phase**

Between conditions

1st 200 m	2nd 200 m	3rd 200 m	4th 200m	5th 200m	6th 200m	Average of first 6 200m
$H_{(12,2)}$=1.038 p=.595	$H_{(12,2)}$=0.115 p=.944	$H_{(12,2)}$=1.500 p=.472	$H_{(12,2)}$=0.500 p=.779	$H_{(12,2)}$=0.808 p=.668	$H_{(12,2)}$=1.654 p=.437	$H_{(12,2)}$=0.731 p=.694

Within condition

Documentary	Smartphone	AX-CPT
$X^2_{(4,6)}$= 2.000 p=.849 W=.100	$X^2_{(4,5)}$= 3.489 p=.625 W=.174	$X^2_{(4,5)}$=4.857 p=.434 W=.243

- **Exit phase**

Between conditions

1st 200 m	2nd 200 m	3rd 200 m	4th 200m	5th 200m	6th 200m	Average of first 6 200m
$H_{(12,2)}$=2.577 p=.276	$H_{(12,2)}$=2.346 p=.309	$H_{(12,2)}$=1.573 p=.455	$H_{(12,2)}$=2.923 p=.232	$H_{(12,2)}$=0.502 p=.778	$H_{(12,2)}$=2.462 p=.292	$H_{(12,2)}$=1.885 p=.390

Within condition

Documentary	Smartphone	AX-CPT
$X^2_{(4,6)}$= 12.050 p=.034* W=.603	$X^2_{(4,5)}$= 6.429 p=.267 W=.321	$X^2_{(4,5)}$=3.000 p=.700 W=.150

Post-hoc

	1st vs 2nd	1st vs 3rd	1st vs 4th	1st vs 5th	1st vs 6th
Documentary	$Z_{(4)}$=--1.095 p=.273 r=.104	$Z_{(4)}$=-1.826 p=.068 r=.101	$Z_{(4)}$=-0.365 p=.715 r=-.653	$Z_{(4)}$=-0.365 p=.715 r=-.228	$Z_{(4)}$=-0.365 p=.715 r=.282

- **Start of the Breathing action (% of stroke cycle - right)**

Between conditions

1st 200 m	2nd 200 m	3rd 200 m	4th 200m	5th 200m	6th 200m	Average of first 6 200m	7th 200m (maximal)
$H_{(12,2)}$=2.755 p=.252	$H_{(12,2)}$=1.209 p=.546	$H_{(12,2)}$=.700 p=.705	$H_{(12,2)}$=.111 p=.946	$H_{(12,2)}$=.400 p=.819	$H_{(12,2)}$=.244 p=.885	$H_{(12,2)}$=.241 p=.886	$H_{(12,2)}$=1.361 p=.506

Within condition

Documentary	Smartphone	AX-CPT
$X^2_{(4,6)}$=4.571 p=.470 W=.457	$X^2_{(4,5)}$=2.286 p=.808 W=.229	$X^2_{(4,5)}$=8.905 p=.113 W=.594

- **Start of the Breathing action (% of stroke cycle – left)**

Between conditions

1st 200 m	2nd 200 m	3rd 200 m	4th 200m	5th 200m	6th 200m	Average of first 6 200m	7th 200m (maximal)
$H_{(12,2)}$=.931 p=.628	$H_{(12,2)}$=1.442 p=.486	$H_{(12,2)}$=.053 p=.974	$H_{(12,2)}$=1.295 p=.523	$H_{(12,2)}$=.326 p=.850	$H_{(12,2)}$=.348 p=.840	$H_{(12,2)}$=2.163 p=.339	$H_{(12,2)}$=.482 p=.786

Within condition

Documentary	Smartphone	AX-CPT
$X^2_{(4,6)}$=3.286 p=.656 W=.164	$X^2_{(4,5)}$=2.286 p=.808 W=.229	$X^2_{(4,5)}$=8.524 p=.130 W=.568

- **End of the Breathing action (% of stroke cycle - right)**

Between conditions

1st 200 m	2nd 200 m	3rd 200 m	4th 200m	5th 200m	6th 200m	Average of first 6 200m	7th 200m (maximal)
$H_{(12,2)}$=3.823 p=.148	$H_{(12,2)}$=1.209 p=.546	$H_{(12,2)}$=.118 p=.943	$H_{(12,2)}$=.244 p=.885	$H_{(12,2)}$=.778 p=.678	$H_{(12,2)}$=.078 p=.962	$H_{(12,2)}$=.173 p=.917	$H_{(12,2)}$=.250 p=.882

Within condition

Documentary	Smartphone	AX-CPT
$X^2_{(4,6)}$=.286 p=.998 W=.029	$X^2_{(4,5)}$=7.429 p=.191 W=.743	$X^2_{(4,5)}$=7.762 p=.170 W=.517

- **End of the Breathing action (% of stroke cycle - left)**

Between conditions

1st 200 m	2nd 200 m	3rd 200 m	4th 200m	5th 200m	6th 200m	Average of first 6 200m	7th 200m (maximal)
$H_{(12,2)}$=.931 p=.628	$H_{(12,2)}$=1.442 p=.486	$H_{(12,2)}$=.326 p=.850	$H_{(12,2)}$=.932 p=.628	$H_{(12,2)}$=1.053 p=.591	$H_{(12,2)}$=.667 p=.717	$H_{(12,2)}$=1.802 p=.406	$H_{(12,2)}$=3.073 p=.215

Within condition

Documentary	Smartphone	AX-CPT
$X^2_{(4,6)}$=5.143 p=.399 W=.257	$X^2_{(4,5)}$=6.571 p=.255 W=.657	$X^2_{(4,5)}$=4.143 p=.529 W=.276

- **First right Kick (% of stroke cycle - right)**

Between conditions

1st 200 m	2nd 200 m	3rd 200 m	4th 200m	5th 200m	6th 200m	Average of first 6 200m	7th 200m (maximal)
$H_{(12,2)}$=1.299 p=.522	$H_{(12,2)}$=1.038 p=.595	$H_{(12,2)}$=.138 p=.933	$H_{(12,2)}$=.191 p=.909	$H_{(12,2)}$=2.251 p=.325	$H_{(12,2)}$=1.615 p=.446	$H_{(12,2)}$=.371 p=.831	$H_{(12,2)}$=.600 p=.741

Within condition

Documentary	Smartphone	AX-CPT
$X^2_{(4,6)}$=7.714 p=.173 W=.771	$X^2_{(4,5)}$=1.714 p=.887 W=.171	$X^2_{(4,5)}$=3.762 p=.584 W=.251

- **First right Kick (% of stroke cycle - left)**

Between conditions

1st 200 m	2nd 200 m	3rd 200 m	4th 200m	5th 200m	6th 200m	Average of first 6 200m	7th 200m (maximal)
$H_{(12,2)}$=.006 p=.997	$H_{(12,2)}$=.132 p=.936	$H_{(12,2)}$=.030 p=.985	$H_{(12,2)}$=.154 p=.926	$H_{(12,2)}$=.029 p=.985	$H_{(12,2)}$=.081 p=.960	$H_{(12,2)}$=.260 p=.878	$H_{(12,2)}$=.167 p=.920

Within condition

Documentary	Smartphone	AX-CPT
$X^2_{(4,6)}$=5.857 p=.320 W=.293	$X^2_{(4,5)}$=9.143 p=.103 W=.914	$X^2_{(4,5)}$=5.095 p=.404 W=.340

- **Second right Kick (% of stroke cycle - right)**

Between conditions

1st 200 m	2nd 200 m	3rd 200 m	4th 200m	5th 200m	6th 200m	Average of first 6 200m	7th 200m (maximal)
$H_{(12,2)}$=030 p=.985	$H_{(12,2)}$=1.846 p=.397	$H_{(12,2)}$=.081 p=.960	$H_{(12,2)}$=1.173 p=.556	$H_{(12,2)}$=1.178 p=.555	$H_{(12,2)}$=3.942 p=.139	$H_{(12,2)}$=.020 p=.990	$H_{(12,2)}$=.700 p=.705

Within condition

Documentary	Smartphone	AX-CPT
$X^2_{(4,6)}$=2.857 p=.722 W=.286	$X^2_{(4,5)}$=1.714 p=.887 W=.171	$X^2_{(4,5)}$=6.238 p=.284 W=.416

- **Second right Kick (% of stroke cycle - left)**

Between conditions

1st 200 m	2nd 200 m	3rd 200 m	4th 200m	5th 200m	6th 200m	Average of first 6 200m	7th 200m (maximal)
$H_{(12,2)}$=.180 p=.914	$H_{(12,2)}$=.119 p=.942	$H_{(12,2)}$=.475 p=.789	$H_{(12,2)}$=.115 p=.944	$H_{(12,2)}$=.542 p=.762	$H_{(12,2)}$=.799 p=.671	$H_{(12,2)}$=1.040 p=.595	$H_{(12,2)}$=.144 p=.931

Within condition

Documentary	Smartphone	AX-CPT
$X^2_{(4,6)}$=2.286 p=.808 W=.114	$X^2_{(4,5)}$=5.429 p=.366 W=.543	$X^2_{(4,5)}$=3.952 p=.556 W=.263

- **Third right Kick (% of stroke cycle - right)**

Between conditions

1st 200 m	2nd 200 m	3rd 200 m	4th 200m	5th 200m	6th 200m	Average of first 6 200m	7th 200m (maximal)
$H_{(12,2)}$=.119 p=.942	$H_{(12,2)}$=1.846 p=.397	$H_{(12,2)}$=1.599 p=.450	$H_{(12,2)}$=.055 p=.973	$H_{(12,2)}$=.364 p=.834	$H_{(12,2)}$=.622 p=.733	$H_{(12,2)}$=.771 p=.680	$H_{(12,2)}$=3.100 p=.212

Within condition

Documentary	Smartphone	AX-CPT
$X^2_{(4,6)}$=5.714 p=.335 W=.571	$X^2_{(4,5)}$=7.143 p=.210 W=.714	$X^2_{(4,5)}$=7.762 p=.170 W=.517

- **Third right Kick (% of stroke cycle - left)**

Between conditions

1st 200 m	2nd 200 m	3rd 200 m	4th 200m	5th 200m	6th 200m	Average of first 6 200m	7th 200m (maximal)
$H_{(12,2)}$=.843 p=.656	$H_{(12,2)}$=1.203 p=.548	$H_{(12,2)}$=.689 p=.709	$H_{(12,2)}$=.269 p=.874	$H_{(12,2)}$=.118 p=.943	$H_{(12,2)}$=.241 p=.886	$H_{(12,2)}$=.620 p=.733	$H_{(12,2)}$=1.076 p=.584

Within condition

Documentary	Smartphone	AX-CPT
$X^2_{(4,6)}$=9.571 p=.088 W=.479	$X^2_{(4,5)}$=3.143 p=.678 W=.314	$X^2_{(4,5)}$=3.000 p=.700 W=.200

- **Duration of first right Kick (% of stroke cycle - right)**

Between conditions

1st 200 m	2nd 200 m	3rd 200 m	4th 200m	5th 200m	6th 200m	Average of first 6 200m	7th 200m (maximal)
$H_{(12,2)}$=1.190 p=.552	$H_{(12,2)}$=.731 p=.694	$H_{(12,2)}$=2.829 p=.243	$H_{(12,2)}$=.736 p=.692	$H_{(12,2)}$=.636 p=.727	$H_{(12,2)}$=.724 p=.696	$H_{(12,2)}$=.131 p=.936	$H_{(12,2)}$=2.500 p=.287

Within condition

Documentary	Smartphone	AX-CPT
$X^2_{(4,6)}$=6.571 p=.255 W=.657	$X^2_{(4,5)}$=4.286 p=.509 W=.429	$X^2_{(4,5)}$=8.333 p=.139 W=.556

- **Duration of first right Kick (% of stroke cycle - left)**

Between conditions

1st 200 m	2nd 200 m	3rd 200 m	4th 200m	5th 200m	6th 200m	Average of first 6 200m	7th 200m (maximal)
$H_{(12,2)}$=.326 p=.850	$H_{(12,2)}$=.475 p=.789	$H_{(12,2)}$=.033 p=.984	$H_{(12,2)}$=.038 p=.981	$H_{(12,2)}$=.214 p=.898	$H_{(12,2)}$=.214 p=.989	$H_{(12,2)}$=.140 p=.932	$H_{(12,2)}$=.053 p=.974

Within condition

Documentary	Smartphone	AX-CPT
$X^2_{(4,6)}$=12.143 p=.033* W=.607	$X^2_{(4,5)}$=5.143 p=.399 W=.514	$X^2_{(4,5)}$=4.714 p=.452 W=.314

Post-hoc

	1 - 2	1 - 3	1 - 4	1 - 5	1 - 6
Documentary	$Z_{(4)}$=-2.023 p=.043*	$Z_{(4)}$=-1.826 p=.068	$Z_{(4)}$=-1.826 p=.068	$Z_{(4)}$=-1.826 p=.068	$Z_{(4)}$=-1.826 p=.068

- **Duration of second right Kick (% of stroke cycle - right)**

Between conditions

1st 200 m	2nd 200 m	3rd 200 m	4th 200m	5th 200m	6th 200m	Average of first 6 200m	7th 200m (maximal)
$H_{(12,2)}$=1.404 p=.495	$H_{(12,2)}$=.808 p=.668	$H_{(12,2)}$=2.472 p=.291	$H_{(12,2)}$=.218 p=.897	$H_{(12,2)}$=.636 p=.727	$H_{(12,2)}$=.622 p=.733	$H_{(12,2)}$=.706 p=.703	$H_{(12,2)}$=.700 p=.705

Within condition

Documentary	Smartphone	AX-CPT
$X^2_{(4,6)}$=4.286 p=.509 W=.429	$X^2_{(4,5)}$=3.429 p=.634 W=.343	$X^2_{(4,5)}$=10.238 p=.069 W=.683

- **Duration of second right Kick (% of stroke cycle - left)**

Between conditions

1^	2^	3^	4^	5^	6^	Avg.	7^
$H_{(12,2)}$=1.046 p=.593	$H_{(12,2)}$=.119 p=.942	$H_{(12,2)}$=.376 p=.829	$H_{(12,2)}$=1.077 p=.584	$H_{(12,2)}$=.241 p=.886	$H_{(12,2)}$=.645 p=.724	$H_{(12,2)}$=.240 p=.887	$H_{(12,2)}$=.045 p=.978

Within condition

Documentary	Smartphone	AX-CPT
$X^2_{(4,6)}$=.857 p=.973 W=.043	$X^2_{(4,5)}$=8.000 p=.156 W=.800	$X^2_{(4,5)}$=5.286 p=.382 W=.352

- **Duration of third right Kick (% of stroke cycle - right)**

Between conditions

1st 200 m	2nd 200 m	3rd 200 m	4th 200m	5th 200m	6th 200m	Average of first 6 200m	7th 200m (maximal)
$H_{(12,2)}$=1.203 p=.548	$H_{(12,2)}$=1.500 p=.472	$H_{(12,2)}$=1.442 p=.486	$H_{(12,2)}$=.736 p=.692	$H_{(12,2)}$=.636 p=.727	$H_{(12,2)}$=.724 p=.696	$H_{(12,2)}$=1.003 p=.606	$H_{(12,2)}$=3.244 p=.197

Within condition

Documentary	Smartphone	AX-CPT
$X^2_{(4,6)}$=4.571 p=.470 W=.457	$X^2_{(4,5)}$=5.429 p=.366 W=.543	$X^2_{(4,5)}$=5.095 p=.404 W=.340

- **Duration of third right Kick (% of stroke cycle - left)**

Between conditions

1st 200 m	2nd 200 m	3rd 200 m	4th 200m	5th 200m	6th 200m	Average of first 6 200m	7th 200m (maximal)
$H_{(12,2)}$=.751 p=.687	$H_{(12,2)}$=.218 p=.897	$H_{(12,2)}$=.000 p=1.000	$H_{(12,2)}$=1.192 p=.551	$H_{(12,2)}$=.138 p=.933	$H_{(12,2)}$=.092 p=.955	$H_{(12,2)}$=.380 p=.827	$H_{(12,2)}$=1.295 p=.523

Within condition

Documentary	Smartphone	AX-CPT
$X^2_{(4,6)}$=9.286 p=.098 W=.464	$X^2_{(4,5)}$=1.429 p=.921 W=.143	$X^2_{(4,5)}$=.905 p=.970 W=.060

- **First left Kick (% of stroke cycle - right)**

Between conditions

1st 200 m	2nd 200 m	3rd 200 m	4th 200m	5th 200m	6th 200m	Average of first 6 200m	7th 200m (maximal)
$H_{(12,2)}$=1.378 p=.502	$H_{(12,2)}$=1.333 p=.513	$H_{(12,2)}$=.344 p=.842	$H_{(12,2)}$=.696 p=.706	$H_{(12,2)}$=.286 p=.867	$H_{(12,2)}$=.238 p=.888	$H_{(12,2)}$=.900 p=.638	$H_{(12,2)}$=.095 p=.953

Within condition

Documentary	Smartphone	AX-CPT
$X^2_{(4,6)}$= p=. W=.	$X^2_{(4,5)}$=3.381 p=.641 W=.225	$X^2_{(4,5)}$= p=. W=.

- **First left Kick (% of stroke cycle - left)**

Between conditions

1st 200 m	2nd 200 m	3rd 200 m	4th 200m	5th 200m	6th 200m	Average of first 6 200m	7th 200m (maximal)
$H_{(12,2)}$=.018 p=.991	$H_{(12,2)}$=1.611 p=.447	$H_{(12,2)}$=482 p=.786	$H_{(12,2)}$=1.178 p=.555	$H_{(12,2)}$=1.867 p=.393	$H_{(12,2)}$=.694 p=.707	$H_{(12,2)}$=.118 p=.943	$H_{(12,2)}$=3.139 p=.208

Within condition

Documentary	Smartphone	AX-CPT
$X^2_{(4,6)}$=4.905 p=.428 W=.327	$X^2_{(4,5)}$=3.952 p=.556 W=.263	$X^2_{(4,5)}$= p=. W=.

- **Second left Kick (% of stroke cycle - right)**

Between conditions

1st 200 m	2nd 200 m	3rd 200 m	4th 200m	5th 200m	6th 200m	Average of first 6 200m	7th 200m (maximal)
$H_{(12,2)}$=.811 p=.667	$H_{(12,2)}$=.667 p=.717	$H_{(12,2)}$=1.778 p=.411	$H_{(12,2)}$=.643 p=.725	$H_{(12,2)}$=.571 p=.751	$H_{(12,2)}$=.857 p=.651	$H_{(12,2)}$=1.000 p=.607	$H_{(12,2)}$=.857 p=.651

Within condition

Documentary	Smartphone	AX-CPT
$X^2_{(4,6)}$= p=. W=.	$X^2_{(4,5)}$=2.048 p=.843 W=.137	$X^2_{(4,5)}$= p=. W=.

- **Second left Kick (% of stroke cycle - left)**

Between conditions

1st 200 m	2nd 200 m	3rd 200 m	4th 200m	5th 200m	6th 200m	Average of first 6 200m	7th 200m (maximal)
$H_{(12,2)}$=3.45 p=.841	$H_{(12,2)}$=2.211 p=.331	$H_{(12,2)}$=.336 p=.845	$H_{(12,2)}$=.444 p=.801	$H_{(12,2)}$=1.156 p=.561	$H_{(12,2)}$=4.028 p=.133	$H_{(12,2)}$=.891 p=.641	$H_{(12,2)}$=1.806 p=.405

Within condition

Documentary	Smartphone	AX-CPT
$X^2_{(4,6)}$=2.619 p=.758 W=.175	$X^2_{(4,5)}$=2.238 p=.815 W=.149	$X^2_{(4,5)}$= p=. W=.

- **Third left Kick (% of stroke cycle - right)**

Between conditions

1st 200 m	2nd 200 m	3rd 200 m	4th 200m	5th 200m	6th 200m	Average of first 6 200m	7th 200m (maximal)
$H_{(12,2)}$=1.344 p=.511	$H_{(12,2)}$=1.000 p=.607	$H_{(12,2)}$=.111 p=.946	$H_{(12,2)}$=.161 p=.923	$H_{(12,2)}$=1.571 p=.456	$H_{(12,2)}$=2.381 p=.304	$H_{(12,2)}$=.600 p=.741	$H_{(12,2)}$=.857 p=.651

Within condition

Documentary	Smartphone	AX-CPT
$X^2_{(4,6)}$= p=. W=.	$X^2_{(4,5)}$=5.857 p=.320 W=.390	$X^2_{(4,5)}$= p=. W=.

- **Third left Kick (% of stroke cycle - left)**

Between conditions

1st 200 m	2nd 200 m	3rd 200 m	4th 200m	5th 200m	6th 200m	Average of first 6 200m	7th 200m (maximal)
$H_{(12,2)}$=.700 p=.705	$H_{(12,2)}$=1.378 p=.502	$H_{(12,2)}$=.636 p=.727	$H_{(12,2)}$=.311 p=.856	$H_{(12,2)}$=.356 p=.837	$H_{(12,2)}$=.472 p=.790	$H_{(12,2)}$=.455 p=.797	$H_{(12,2)}$=.556 p=.757

Within condition

Documentary	Smartphone	AX-CPT
$X^2_{(4,6)}$=.905 p=.970 W=.060	$X^2_{(4,5)}$=.905 p=.970 W=.060	$X^2_{(4,5)}$= p=. W=.

- **Duration of first left Kick (% of stroke cycle - right)**

Between conditions

1st 200 m	2nd 200 m	3rd 200 m	4th 200m	5th 200m	6th 200m	Average of first 6 200m	7th 200m (maximal)
$H_{(12,2)}$=.444<br>p=.801	$H_{(12,2)}$=.500<br>p=.779	$H_{(12,2)}$=.000<br>p=.1.000	$H_{(12,2)}$=.268<br>p=..875	$H_{(12,2)}$=.000<br>p=.1.000	$H_{(12,2)}$=.238<br>p=.888	$H_{(12,2)}$=.244<br>p=.885	$H_{(12,2)}$=.238<br>p=.888

Within condition

Documentary	Smartphone	AX-CPT
$X^2_{(4,6)}$=<br>p=.<br>W=.	$X^2_{(4,5)}$=6.619<br>p=.251<br>W=.441	$X^2_{(4,5)}$=<br>p=.<br>W=.

- **Duration of first left Kick (% of stroke cycle - left)**

Between conditions

1st 200 m	2nd 200 m	3rd 200 m	4th 200m	5th 200m	6th 200m	Average of first 6 200m	7th 200m (maximal)
$H_{(12,2)}$=1.209<br>p=.546	$H_{(12,2)}$=.811<br>p=.667	$H_{(12,2)}$=.482<br>p=.786	$H_{(12,2)}$=.900<br>p=.638	$H_{(12,2)}$=.267<br>p=.875	$H_{(12,2)}$=.694<br>p=.707	$H_{(12,2)}$=.482<br>p=.786	$H_{(12,2)}$=.556<br>p=.757

Within condition

Documentary	Smartphone	AX-CPT
$X^2_{(4,6)}$=2-238<br>p=.815<br>W=.149	$X^2_{(4,5)}$=3.952<br>p=.556<br>W=.263	$X^2_{(4,5)}$=<br>p=.<br>W=.

- **Duration of second left Kick (% of stroke cycle - right)**

Between conditions

1st 200 m	2nd 200 m	3rd 200 m	4th 200m	5th 200m	6th 200m	Average of first 6 200m	7th 200m (maximal)
$H_{(12,2)}$=2.144 p=.342	$H_{(12,2)}$=1.125 p=.570	$H_{(12,2)}$=.900 p=.638	$H_{(12,2)}$=.321 p=.852	$H_{(12,2)}$=1.286 p=.526	$H_{(12,2)}$=.429 p=.807	$H_{(12,2)}$=1.611 p=.447	$H_{(12,2)}$=3.524 p=.172

Within condition

Documentary	Smartphone	AX-CPT
$X^2_{(4,6)}$= p=. W=.	$X^2_{(4,5)}$=1.286 p=.936 W=.086	$X^2_{(4,5)}$= p=. W=.

- **Duration of second left Kick (% of stroke cycle - left)**

Between conditions

1st 200 m	2nd 200 m	3rd 200 m	4th 200m	5th 200m	6th 200m	Average of first 6 200m	7th 200m (maximal)
$H_{(12,2)}$=.891 p=.641	$H_{(12,2)}$=.244 p=.885	$H_{(12,2)}$=.118 p=.943	$H_{(12,2)}$=2.211 p=.331	$H_{(12,2)}$=.622 p=.733	$H_{(12,2)}$=.556 p=.757	$H_{(12,2)}$=.482 p=.786	$H_{(12,2)}$=1.111 p=.574

Within condition

Documentary	Smartphone	AX-CPT
$X^2_{(4,6)}$=7.381 p=.194 W=.492	$X^2_{(4,5)}$=4.333 p=.502 W=.289	$X^2_{(4,5)}$= p=. W=.

- **Duration of second left Kick (% of stroke cycle - right)**

Between conditions

1st 200 m	2nd 200 m	3rd 200 m	4th 200m	5th 200m	6th 200m	Average of first 6 200m	7th 200m (maximal)
$H_{(12,2)}$=.111 p=.946	$H_{(12,2)}$=1.125 p=.570	$H_{(12,2)}$=.278 p=.870	$H_{(12,2)}$=.643 p=.725	$H_{(12,2)}$=.143 p=.931	$H_{(12,2)}$=.238 p=.888	$H_{(12,2)}$=.000 p=.1.000	$H_{(12,2)}$=.429 p=.807

Within condition

Documentary	Smartphone	AX-CPT
$X^2_{(4,6)}$= p=. W=.	$X^2_{(4,5)}$=6.048 p=.302 W=.403	$X^2_{(4,5)}$= p=. W=.

- **Duration of third left Kick (% of stroke cycle - left)**

Between conditions

1st 200 m	2nd 200 m	3rd 200 m	4th 200m	5th 200m	6th 200m	Average of first 6 200m	7th 200m (maximal)
$H_{(12,2)}$=1.427 p=.490	$H_{(12,2)}$=.100 p=.951	$H_{(12,2)}$=2.091 p=.352	$H_{(12,2)}$=.978 p=.613	$H_{(12,2)}$=.978 p=.613	$H_{(12,2)}$=.472 p=.790	$H_{(12,2)}$=.018 p=.991	$H_{(12,2)}$=.222 p=.895

Within condition

Documentary	Smartphone	AX-CPT
$X^2_{(4,6)}$=5.476 p=.361 W=.365	$X^2_{(4,5)}$=.905 p=.970 W=.060	$X^2_{(4,5)}$= p=. W=.

- **Left hand entry (% of stroke cycle - right)**

Between conditions

1st 200 m	2nd 200 m	3rd 200 m	4th 200m	5th 200m	6th 200m	Average of first 6 200m	7th 200m (maximal)
$H_{(12,2)}$=.086 p=.958	$H_{(12,2)}$=.267 p=.875	$H_{(12,2)}$=.220 p=.896	$H_{(12,2)}$=1.200 p=.549	$H_{(12,2)}$=.723 p=.697	$H_{(12,2)}$=.041 p=.980	$H_{(12,2)}$=.771 p=.680	$H_{(12,2)}$=.082 p=.960

Within condition

Documentary	Smartphone	AX-CPT
$X^2_{(4,6)}$=8.000 p=.156 W=.800	$X^2_{(4,5)}$=10.429 p=.064 W=.695	$X^2_{(4,5)}$=7.951 p=.159 W=.530

- **Right hand entry (% of stroke cycle - left)**

Between conditions

1st 200 m	2nd 200 m	3rd 200 m	4th 200m	5th 200m	6th 200m	Average of first 6 200m	7th 200m (maximal)
$H_{(12,2)}$=.903 p=.637	$H_{(12,2)}$=.096 p=.953	$H_{(12,2)}$=.422 p=.810	$H_{(12,2)}$=1.641 p=.440	$H_{(12,2)}$=.273 p=.873	$H_{(12,2)}$=.164 p=.921	$H_{(12,2)}$=.360 p=.835	$H_{(12,2)}$=.191 p=.909

Within condition

Documentary	Smartphone	AX-CPT
$X^2_{(4,6)}$=8.286 p=.141 W=.829	$X^2_{(4,5)}$=8.714 p=.121 W=.581	$X^2_{(4,5)}$=9.286 p=.098 W=.619

- **Kick rate - right**

Between conditions

1st 200 m	2nd 200 m	3rd 200 m	4th 200m	5th 200m	6th 200m	Average of first 6 200m	7th 200m (maximal)
$H_{(12,2)}$=.203 p=.904	$H_{(12,2)}$=1.530 p=.465	$H_{(12,2)}$=.382 p=.825	$H_{(12,2)}$=.808 p=.668	$H_{(12,2)}$=.241 p=.886	$H_{(12,2)}$=.081 p=.960	$H_{(12,2)}$=.780 p=.677	$H_{(12,2)}$=.144 p=.931

Within condition

Documentary	Smartphone	AX-CPT
$X^2_{(4,6)}$=3.857 p=.570 W=.193	$X^2_{(4,5)}$=8.857 p=.115 W=.886	$X^2_{(4,5)}$=13.667 p=.018* W=.911

Post-hoc

	1 - 2	1 - 3	1 - 4	1 - 5	1 - 6
AX-CPT	$Z_{(4)}$=-.730 p=.465	$Z_{(4)}$=-.944 p=.345	$Z_{(4)}$=-1.095 p=.273	$Z_{(4)}$=-1.483 p=.138	$Z_{(4)}$=-2.023 p=.043*

- **Kick rate - left**

Between conditions

1st 200 m	2nd 200 m	3rd 200 m	4th 200m	5th 200m	6th 200m	Average of first 6 200m	7th 200m (maximal)
$H_{(12,2)}$=1.064 p=.588	$H_{(12,2)}$=1.611 p=.447	$H_{(12,2)}$=.636 p=.727	$H_{(12,2)}$=.000 p=.1.000	$H_{(12,2)}$=1.689 p=.430	$H_{(12,2)}$=1.806 p=.405	$H_{(12,2)}$=1.209 p=.546	$H_{(12,2)}$=2.778 p=.249

Within condition

Documentary	Smartphone	AX-CPT
$X^2_{(4,6)}$=7.571 p=.181 W=.505	$X^2_{(4,5)}$=5.095 p=.404 W=.340	$X^2_{(4,5)}$= p=. W=.

- **Kick time - right**

Between conditions

1st 200 m	2nd 200 m	3rd 200 m	4th 200m	5th 200m	6th 200m	Average of first 6 200m	7th 200m (maximal)
$H_{(12,2)}$=.386 p=.825	$H_{(12,2)}$=2.047 p=.359	$H_{(12,2)}$=.940 p=.625	$H_{(12,2)}$=3.115 p=.211	$H_{(12,2)}$=1.718 p=.424	$H_{(12,2)}$=1.106 p=.575	$H_{(12,2)}$=.780 p=.677	$H_{(12,2)}$=2.053 p=.358

Within condition

Documentary	Smartphone	AX-CPT
$X^2_{(4,6)}$=3.571 p=.613 W=.179	$X^2_{(4,5)}$=8.857 p=.115 W=.886	$X^2_{(4,5)}$=11.190 p=.048* W=.746

Post-hoc

	1st vs 2nd	1st vs 3rd	1st vs 4th	1st vs 5th	1st vs 6th
AX-CPT	$Z_{(4)}$=-1.461 p=.144	$Z_{(4)}$=-2.023 p=.043*	$Z_{(4)}$=-1.461 p=.144	$Z_{(4)}$=-1.753 p=.080	$Z_{(4)}$=-1.753 p=.080

- **Kick time - left**

Between conditions

1st 200 m	2nd 200 m	3rd 200 m	4th 200m	5th 200m	6th 200m	Average of first 6 200m	7th 200m (maximal)
$H_{(12,2)}$=1.073 p=.585	$H_{(12,2)}$=1.944 p=.378	$H_{(12,2)}$=1.573 p=.455	$H_{(12,2)}$=4.278 p=.118	$H_{(12,2)}$=.622 p=.733	$H_{(12,2)}$=1.139 p=.566	$H_{(12,2)}$=2.955 p=.228	$H_{(12,2)}$=2.472 p=.291

Within condition

Documentary	Smartphone	AX-CPT
$X^2_{(4,6)}$=8.524<br>p=.130<br>W=.568	$X^2_{(4,5)}$=3.190<br>p=.671<br>W=.213	$X^2_{(4,5)}$=<br>p=.<br>W=.

- **Stroke time - right**

Between conditions

1st 200 m	2nd 200 m	3rd 200 m	4th 200m	5th 200m	6th 200m	Average of first 6 200m	7th 200m (maximal)
$H_{(12,2)}$=1.958<br>p=.376	$H_{(12,2)}$=.399<br>p=.819	$H_{(12,2)}$=.563<br>p=.755	$H_{(12,2)}$=.864<br>p=.649	$H_{(12,2)}$=.541<br>p=.763	$H_{(12,2)}$=.564<br>p=.754	$H_{(12,2)}$=.203<br>p=.904	$H_{(12,2)}$=.191<br>p=.909

Within condition

Documentary	Smartphone	AX-CPT
$X^2_{(4,6)}$=7.714<br>p=.173<br>W=.771	$X^2_{(4,5)}$=11.381<br>p=.044*<br>W=.759	$X^2_{(4,5)}$=12.143<br>p=.033*<br>W=.810

Post-hoc

	1st vs 2nd	1st vs 3rd	1st vs 4th	1st vs 5th	1st vs 6th
Smartphone	$Z_{(4)}$=-.730<br>p=.465	$Z_{(4)}$=-1.214<br>p=.225	$Z_{(4)}$=-1.826<br>p=.068	$Z_{(4)}$=-1.753<br>p=.080	$Z_{(4)}$=-1.753<br>p=.080

- **Stroke time - left**

Between conditions

1st 200 m	2nd 200 m	3rd 200 m	4th 200m	5th 200m	6th 200m	Average of first 6 200m	7th 200m (maximal)
$H_{(12,2)}$=1.286 p=.526	$H_{(12,2)}$=.726 p=.696	$H_{(12,2)}$=.280 p=.869	$H_{(12,2)}$=.870 p=.647	$H_{(12,2)}$=.086 p=.958	$H_{(12,2)}$=.119 p=.942	$H_{(12,2)}$=.560 p=.756	$H_{(12,2)}$=.115 p=.944

Within condition

Documentary	Smartphone	AX-CPT
$X^2_{(4,6)}$=15.286 p=.009* W=.764	$X^2_{(4,5)}$=11.952 p=.035* W=.797	$X^2_{(4,5)}$=11.571 p=.041* W=.771

Post-hoc

	1 - 2	1 - 3	1 - 4	1 - 5	1 - 6
Documentary	$Z_{(4)}$=-1.753 p=.080	$Z_{(4)}$=-1.826 p=.068	$Z_{(4)}$=-1.826 p=.068	$Z_{(4)}$=-1.826 p=.068	$Z_{(4)}$=-1.826 p=.068
Smartphone	$Z_{(4)}$=-.730 p=.465	$Z_{(4)}$=-1.095 p=.273	$Z_{(4)}$=-1.095 p=.273	$Z_{(4)}$=-1.604 p=.109	$Z_{(4)}$=-1.604 p=.109
AX-CPT	$Z_{(4)}$=-.730 p=.465	$Z_{(4)}$=-1.214 p=.225	$Z_{(4)}$=-1.461 p=.144	$Z_{(4)}$=-1.013 p=.043*	$Z_{(4)}$=-1.013 p=.043*

- **Number of right strokes for each length**

Between conditions

1st 200 m	2nd 200 m	3rd 200 m	4th 200m	5th 200m	6th 200m	Average of first 6 200m	7th 200m (maximal)
$H_{(12,2)}$=.257 p=.879	$H_{(12,2)}$=.536 p=.765	$H_{(12,2)}$=.368 p=.832	$H_{(12,2)}$=.542 p=.763	$H_{(12,2)}$=1.417 p=.492	$H_{(12,2)}$=1.398 p=.497	$H_{(12,2)}$=.326 p=.850	$H_{(12,2)}$=3.855 p=.145

Within condition

Documentary	Smartphone	AX-CPT

$X^2_{(4,6)}$=6.983<br>p=.222<br>W=.698	$X^2_{(4,5)}$= 10.825<br>p=.055<br>W=.722	$X^2_{(4,5)}$=12.551<br>p=.028*<br>W=.837

Post-hoc

	1 - 2	1 - 3	1 - 4	1 - 5	1 - 6
AX-CPT	$Z_{(4)}$=-1.841<br>p=.066	$Z_{(4)}$=-2.023<br>p=.043*	$Z_{(4)}$=-1.826<br>p=.068	$Z_{(4)}$=-2.032<br>p=.042*	$Z_{(4)}$=-2.023<br>p=.043*

- **Number of left strokes for each length**

Between conditions

1st 200 m	2nd 200 m	3rd 200 m	4th 200m	5th 200m	6th 200m	Average of first 6 200m	7th 200m (maximal)
$H_{(12,2)}$=.082<br>p=.960	$H_{(12,2)}$=.380<br>p=.827	$H_{(12,2)}$=.724<br>p=.696	$H_{(12,2)}$=.380<br>p=.827	$H_{(12,2)}$=1.020<br>p=.600	$H_{(12,2)}$=.751<br>p=.687	$H_{(12,2)}$=.246<br>p=.884	$H_{(12,2)}$=2.101<br>p=.350

Within condition

Documentary	Smartphone	AX-CPT
$X^2_{(4,6)}$=14.147<br>p=.015*<br>W=.707	$X^2_{(4,5)}$=7.990<br>p=.157<br>W=.533	$X^2_{(4,5)}$=13.614<br>p=.018*<br>W=.908

Post-hoc

	1st vs 2nd	1st vs 3rd	1st vs 4th	1st vs 5th	1st vs 6th
Documentary	$Z_{(4)}$=-1.841<br>p=.066	$Z_{(4)}$=-1.604<br>p=.109	$Z_{(4)}$=-1.841<br>p=.066	$Z_{(4)}$=-1.826<br>p=.068	$Z_{(4)}$=-1.826<br>p=.068
AX-CPT	$Z_{(4)}$=-1.826<br>p=.068	$Z_{(4)}$=-1.826<br>p=.068	$Z_{(4)}$=-1.826<br>p=.068	$Z_{(4)}$=-2.023<br>p=.043*	$Z_{(4)}$=-2.023<br>p=.043*

- **Right stroke length**

Between conditions

1st 200 m	2nd 200 m	3rd 200 m	4th 200m	5th 200m	6th 200m	Average of first 6 200m	7th 200m (maximal)
$H_{(12,2)}$=.096 p=.953	$H_{(12,2)}$=.267 p=.875	$H_{(12,2)}$=.457 p=.796	$H_{(12,2)}$=.368 p=.832	$H_{(12,2)}$=.255 p=.880	$H_{(12,2)}$=1.391 p=.499	$H_{(12,2)}$=.523 p=.770	$H_{(12,2)}$=2.373 p=.305

Within condition

Documentary	Smartphone	AX-CPT
$X^2_{(4,6)}$=5.429 p=.366 W=.543	$X^2_{(4,5)}$=9.667 p=.085 W=.644	$X^2_{(4,5)}$=11.952 p=.035* W=.797

Post-hoc

	1st vs 2nd	1st vs 3rd	1st vs 4th	1st vs 5th	1st vs 6th
AX-CPT	$Z_{(4)}$=-1.826 p=.068	$Z_{(4)}$=-2.023 p=.043*	$Z_{(4)}$=-1.826 p=.068	$Z_{(4)}$=-2.023 p=.043*	$Z_{(4)}$=-2.023 p=.043*

- **Left stroke length**

Between conditions

1st 200 m	2nd 200 m	3rd 200 m	4th 200m	5th 200m	6th 200m	Average of first 6 200m	7th 200m (maximal)
$H_{(12,2)}$=.111 p=.946	$H_{(12,2)}$=.771 p=.680	$H_{(12,2)}$=.326 p=.850	$H_{(12,2)}$=.218 p=.897	$H_{(12,2)}$=.030 p=.985	$H_{(12,2)}$=.597 p=.742	$H_{(12,2)}$=.020 p=.990	$H_{(12,2)}$=1.654 p=.437

Within condition

Documentary	Smartphone	AX-CPT
$X^2_{(4,6)}$= 11.00<br>p=.051<br>W=.550	$X^2_{(4,5)}$=7.000<br>p=.221<br>W=.467	$X^2_{(4,5)}$=10.810<br>p=.055<br>W=.721

- **Stroke index (right)**

Between conditions

1st 200 m	2nd 200 m	3rd 200 m	4th 200m	5th 200m	6th 200m	Average of first 6 200m	7th 200m (maximal)
$H_{(12,2)}$=.033<br>p=.984	$H_{(12,2)}$=.475<br>p=.789	$H_{(12,2)}$=.484<br>p=.785	$H_{(12,2)}$=.368<br>p=.832	$H_{(12,2)}$=.255<br>p=.880	$H_{(12,2)}$=.950<br>p=.622	$H_{(12,2)}$=.326<br>p=.850	$H_{(12,2)}$=2.673<br>p=.263

Within condition

Documentary	Smartphone	AX-CPT
$X^2_{(4,6)}$=5.429<br>p=.366<br>W=.543	$X^2_{(4,5)}$=10.619<br>p=.059<br>W=.708	$X^2_{(4,5)}$=10.238<br>p=.069<br>W=.683

- **Stroke index (left)**

Between conditions

1st 200 m	2nd 200 m	3rd 200 m	4th 200m	5th 200m	6th 200m	Average of first 6 200m	7th 200m (maximal)
$H_{(12,2)}$=.043<br>p=.979	$H_{(12,2)}$=.726<br>p=.696	$H_{(12,2)}$=.546<br>p=.761	$H_{(12,2)}$=.119<br>p=.942	$H_{(12,2)}$=.597<br>p=.742	$H_{(12,2)}$=.030<br>p=.985	$H_{(12,2)}$=.000<br>p=.1.000	$H_{(12,2)}$=.808<br>p=.668

Within condition

Documentary	Smartphone	AX-CPT
$X^2_{(4,6)}$=9.00<br>p=.109<br>W=.450	$X^2_{(4,5)}$=8.333<br>p=.139<br>W=.556	$X^2_{(4,5)}$=10.429<br>p=.064<br>W=.695

- **Number of right kicks for each length**

Between conditions

1st 200 m	2nd 200 m	3rd 200 m	4th 200m	5th 200m	6th 200m	Average of first 6 200m	7th 200m (maximal)
$H_{(12,2)}$=1.037<br>p=.595	$H_{(12,2)}$=.267<br>p=.875	$H_{(12,2)}$=.689<br>p=.709	$H_{(12,2)}$=1.885<br>p=.390	$H_{(12,2)}$=.426<br>p=.808	$H_{(12,2)}$=.498<br>p=.780	$H_{(12,2)}$=.320<br>p=.852	$H_{(12,2)}$=5.386<br>p=.068

Within condition

Documentary	Smartphone	AX-CPT
$X^2_{(4,6)}$=3.417<br>p=.636<br>W=.171	$X^2_{(4,5)}$=8.857<br>p=.115<br>W=.886	$X^2_{(4,5)}$=12.233<br>p=.032*<br>W=.816

Post-hoc

	1st vs 2nd	1st vs 3rd	1st vs 4th	1st vs 5th	1st vs 6th
AX-CPT	$Z_{(4)}$=-1.826<br>p=.068	$Z_{(4)}$=-2.023<br>p=.043*	$Z_{(4)}$=-1.826<br>p=.068	$Z_{(4)}$=-2.023<br>p=.043*	$Z_{(4)}$=-1.483<br>p=.138

- **Number of left kicks for each length**

Between conditions

1st 200 m	2nd 200 m	3rd 200 m	4th 200m	5th 200m	6th 200m	Average of first 6 200m	7th 200m (maximal)

$H_{(12,2)}$=.636 p=.727	$H_{(12,2)}$=.227 p=.893	$H_{(12,2)}$=2.200 p=.333	$H_{(12,2)}$=2.378 p=.305	$H_{(12,2)}$=1.867 p=.393	$H_{(12,2)}$=5.361 p=.069	$H_{(12,2)}$=1.064 p=.588	$H_{(12,2)}$=4.250 p=.119

Within condition

Documentary	Smartphone	AX-CPT
$X^2_{(4,6)}$=6.346 p=.274 W=.423	$X^2_{(4,5)}$=5.667 p=.340 W=.378	$X^2_{(4,5)}$= p=. W=.

- **Total number of breathing actions for each length**

Between conditions

1st 200 m	2nd 200 m	3rd 200 m	4th 200m	5th 200m	6th 200m	Average of first 6 200m	7th 200m (maximal)
$H_{(12,2)}$=.122 p=.941	$H_{(12,2)}$=.916 p=.632	$H_{(12,2)}$=.000 p=.1.000	$H_{(12,2)}$=.394 p=.821	$H_{(12,2)}$=.352 p=.839	$H_{(12,2)}$=.584 p=.747	$H_{(12,2)}$=.352 p=.839	$H_{(12,2)}$=1.136 p=.567

Within condition

Documentary	Smartphone	AX-CPT
$X^2_{(4,6)}$=10.591 p=.060 W=.530	$X^2_{(4,5)}$=9.638 p=.086 W=.964	$X^2_{(4,5)}$=11.543 p=.042* W=.770

Post-hoc

	1st vs 2nd	1st vs 3rd	1st vs 4th	1st vs 5th	1st vs 6th
AX-CPT	$Z_{(4)}$=-1.604 p=.109	$Z_{(4)}$=-1.826 p=.068	$Z_{(4)}$=-1.826 p=.068	$Z_{(4)}$=-1.461 p=.144	$Z_{(4)}$=-1.826 p=.068
